# Integrative Approaches to Myopathies and Muscular Dystrophies: Molecular Mechanisms, Diagnostics, and Future Therapies

**DOI:** 10.3390/ijms26167972

**Published:** 2025-08-18

**Authors:** Maja Ziemian, Joanna Szmydtka, Wojciech Snoch, Sandra Milner, Szymon Wojciechowski, Aleksandra Dłuszczakowska, Jakub W. Chojnowski, Zofia Pallach, Katarzyna Żamojda, Grzegorz Węgrzyn, Estera Rintz

**Affiliations:** 1 Department of Molecular Biology, Faculty of Biology, University of Gdańsk, Wita Stwosza 59, 80-308 Gdansk, Poland; m.ziemian.400@studms.ug.edu.pl (M.Z.); j.szmydtka.920@studms.ug.edu.pl (J.S.); wojciech.snoch@ug.edu.pl (W.S.); s.milner.149@studms.ug.edu.pl (S.M.); s.wojciechowski.403@studms.ug.edu.pl (S.W.); a.dluszczakowska.251@studms.ug.edu.pl (A.D.); j.chojnowski.231@studms.ug.edu.pl (J.W.C.); z.pallach.921@studms.ug.edu.pl (Z.P.); k.zamojda.717@studms.ug.edu.pl (K.Ż.); grzegorz.wegrzyn@ug.edu.pl (G.W.); 2 International Centre for Cancer Vaccine Science, University of Gdansk, Kładki 24, 80-822 Gdansk, Poland

**Keywords:** muscular diseases, myopathies, protein quality control, gene therapy, drug repositioning

## Abstract

Myopathies and muscular dystrophies are a diverse group of rare or ultra-rare diseases that significantly impact patients’ quality of life and pose major challenges for diagnosis and treatment. Despite their heterogeneity, many share common molecular mechanisms, particularly involving sarcomeric dysfunction, impaired autophagy, and disrupted gene expression. This review explores the genetic and pathophysiological foundations of major myopathy subtypes, including cardiomyopathies, metabolic and mitochondrial myopathies, congenital and distal myopathies, myofibrillar myopathies, inflammatory myopathies, and muscular dystrophies. Special emphasis is placed on the role of autophagy dysregulation in disease progression, as well as its therapeutic potential. We discuss emerging diagnostic approaches, such as whole-exome sequencing, advanced imaging, and muscle biopsy, alongside therapeutic strategies, including physiotherapy, supplementation, autophagy modulators, and gene therapies. Gene therapy methods, such as adeno-associated virus (AAV) vectors, CRISPR-Cas9, and antisense oligonucleotide, are evaluated for their promise and limitations. The review also highlights the potential of drug repurposing and artificial intelligence tools in advancing diagnostics and personalized treatment. By identifying shared molecular targets, particularly in autophagy and proteostasis networks, we propose unified therapeutic strategies across multiple myopathy subtypes. Finally, we discuss international research collaborations and rare disease programs that are driving innovation in this evolving field.

## 1. Introduction

Myopathies represent a broad group of rare diseases characterized by abnormal development and structural defects in sarcomeres, the fundamental units of muscle fibers. These abnormalities lead to deterioration in both contractile and diastolic parameters, ultimately reducing muscle strength and performance. The genetic basis of myopathies is diverse, with mutations that prominently affect muscle cells, neurons, and may also disrupt connections between them. These diseases significantly reduce patients’ quality of life, often necessitating constant care, and in severe cases, lead to premature death [1,2,3,4,5,6,7,8].

**Figure 1:**
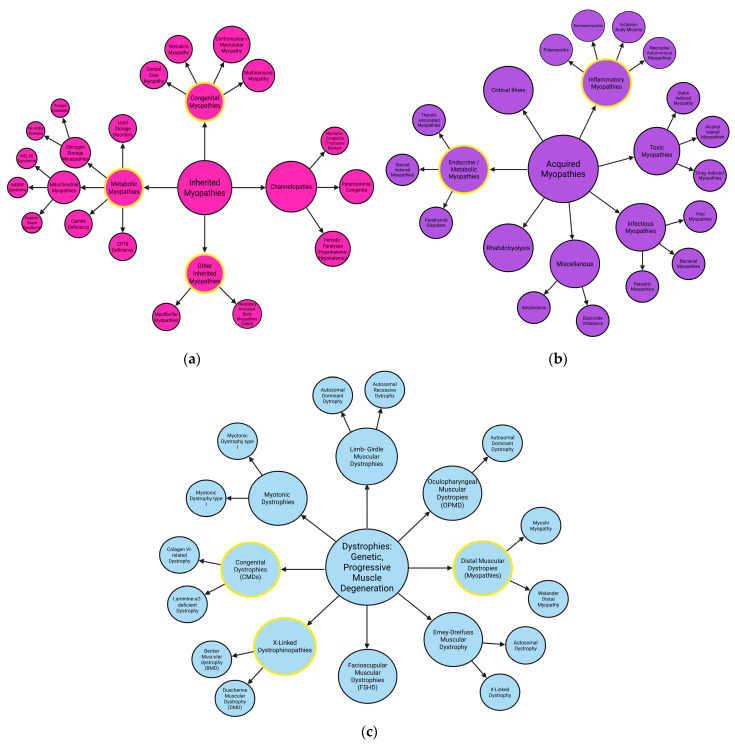
The main classification of myopathies: (**a**) inherited myopathies, (**b**) acquired myopathies, and (**c**) muscular dystrophies; yellow borders point out described in this article. Created in BioRender. Rintz, E. (2025) (**a**) https://BioRender.com/5pub3bg; (**b**) https://BioRender.com/xkfzqrb; (**c**) https://BioRender.com/702uwk4.

More than 50 distinct types of myopathies and muscular dystrophies, have been identified and their types and subtypes are classified into the following main categories: inherited myopathies (congenital metabolic, channelopathies, other inherited myopathies, like myofibrillar myopathies and cardiomyopathies), acquired myopathies (inflammatory myopathies, infectious, toxic, critical illness myopathies, and rhabdobyolysis) and dystrophies (Figure 1) [9,10,11,12,13,14,15]. Their classification depends on the genetic causes, pathomechanisms, and genetic background of a given myopathy. In this article, we decided to focus particularly on those that are related to each other and/or to the disruption of autophagy pathways. We highlighted the details: myofibrillar myopathies, distal myopathies/dystrophies, congenital myopathies, inflammatory myopathies, metabolic myopathies, mitochondrial, cardiomyopathies, and a subtype of the myopathies: muscular dystrophies, which are also the most represented muscular diseases in the human population [9,10,11,12,13,14,15,16]. Estimating the global prevalence of all myopathies is challenging. However, specific diseases have known incidence rates: for example, myofibrillar myopathies affect 1 in 50,000 to 100,000 individuals, distal myopathies 1 in 20,000 to 50,000, congenital myopathies 4–6 per 100,000 live births, idiopathic inflammatory myopathies 9 to 32 per 100,000, metabolic myopathies 1 in 50,000, and cardiomyopathies approximately 1 in 250 individuals [17,18]. Moreover, according to orpha.net, 1–9 cases per 100,000 persons of muscular dystrophies are diagnosed worldwide [19].

Each of these myopathies are rare or ultra-rare, with distinct genetic underpinnings. Consequently, individual conditions often lack commercial appeal for pharmaceutical and biotechnology companies. Additionally, when considering the aggregate burden of all myopathies dystrophies and neuromuscular diseases, the challenge becomes relevant, not only for patients and their families, but also for healthcare systems due to sophisticated and expensive methods of treatment [20]. Identifying commonalities across myopathies and dystrophies could facilitate the development of systemic solutions for therapies, treatment approaches, and funding mechanisms [21,22,23,24,25,26].

The growing awareness of this issue is evident from the increasing number of foundations dedicated to supporting patients. Simultaneously, the global market for neuromuscular disorders is expanding, with more than 15 big companies actively seeking solutions. For instance, according to Transparency Market Research, the market size for idiopathic inflammatory myopathies was estimated at USD 447 million in 2023, with a projected compound annual growth rate (CAGR) of 21.1% from 2024 to 2034. Similarly, the Duchenne muscular dystrophy market size in 2023 was USD 2.2 billion, expected to grow at a CAGR of 11.7%, reaching USD 7.4 billion by 2034. Overall, the neuromuscular disorders market is growing at a CAGR of 10–15%.

This review focuses on the aetiology, progression, and symptomatic treatments for myopathies, emphasizing slowing disease progression. Diagnostic tools, such as muscle biopsy, whole-exome sequencing, nanopore sequencing, high-resolution magnetic resonance imaging (MRI), ultrasound technique and computed tomography (CT) will be discussed [27,28,29,30,31,32]. Additionally, physiotherapeutic techniques to support muscle function will be reviewed [33,34]. Supplementation strategies will be examined for their benefits and risks, particularly those targeting autophagy pathways (p38/MAPK, AMPK/mTOR, FoxO1, SIRT1) [35,36,37,38,39]. The concept of drug repositioning and its application to myopathies will also be addressed. We will discuss how specific drugs or supplements interact with certain signaling pathways and what risks are associated with incorrect dosing, which may cause imbalances and lead to further complications [40,41].

Gene therapies, promising but challenging approaches, will also be explored. Three primary strategies under development will be highlighted: silencing mutated alleles, adding non-mutated or missing alleles, and repairing point mutations through prime and base editing technologies. Delivery mechanisms, such as adeno-associated viruses, lipid nanoparticles, liposomes, and RNA or/and DNA conjugates, will be discussed, with a focus on how their design affects tissue targeting and therapeutic selectivity [42,43,44,45,46,47,48,49,50]. Both symptomatic treatments and gene therapies require rigorous development and testing protocols, from in silico modeling to in vitro, in vivo, and preclinical trials [51,52].

The primary aim of this review is to identify the common features and mechanisms underlying most myopathies and to propose unified treatment strategies. A key focus will be the role of gene expression dysregulation and its impact on autophagy, a process that often becomes imbalanced during disease progression, contributing to the development of secondary myopathies or dystrophies [53,54,55]. Furthermore, the potential of artificial intelligence (AI) tools to aid in diagnosing, monitoring, and treating myopathies will be discussed [56,57]. Finally, the review will highlight rare disease programs and international collaborations aimed at addressing the challenges faced by patients and their families, including funding opportunities and treatment pipelines.

## 2. Myopathies

### 2.1. Cardiomyopathies

Cardiomyopathies are diseases of the heart muscle that affect the structure and function of the organ. According to data provided by the National Heart, Lung, and Blood Institute, eight main types of cardiomyopathies are distinguished. This classification is primarily based on the way the heart is deformed, and how its function is disrupted (e.g., thinning or thickening of the ventricular walls) as well as on specific triggers that may lead to the development of the disease (e.g., severe stress and broken heart syndrome). One of the most common types is dilated cardiomyopathy (DCM). It is estimated that globally, approximately one in every 250 individuals is affected [2,8]. In European pilot studies conducted between 2012 and 2013, 346 cases of DCM were collected [1]. Meanwhile, studies conducted in the U.S. population in 2019 and the prevalence of disease between 2017 and 2019 estimated around 388,350 cases of DCM [3]. However, there is no precise data on the current global prevalence of the disease. Etiology is also not clearly defined, as it can be classified into idiopathic, genetic, or due to various external factors, such as infections caused by pathogens or exposure to toxic substances. However, the most common genetic cause of dilated cardiomyopathy may be caused by mutations in over 50 genes encoding structural proteins of cardiomyocytes (*TTN*, *PLN*, *MYH7*, *BAG3*, etc.) [8].

To present the natural history of DCM, The Multicenter Intervention in Myocarditis and Acute Cardiomyopathy was conducted. The trial focused on idiopathic cardiomyopathy of acute onset. A total of 372 patients with acute dilated cardiomyopathy were enrolled. Mean age of the study cohort was 45 ± 4 years and 38% were female. Mean initial of left ventricular ejection fraction (LVEF) was 24% ± 8%. Baseline therapy included an angiotensin-converting enzyme inhibitor (ACEI) or angiotensin receptor blocker (91%), beta-adrenergic blocker (82%), loop diuretic (67%), and an aldosterone receptor antagonist (27%). Therapy was further intensified at 6 months. The mean LVEF at 6 months increased from 24% ± 8% to 40% ± 12%. LVEF normalized (LVEF > 50%) in 25% of patients. There were 45 hospitalizations for decompensated heart failure (12%) and 17% of patients underwent heart transplantation. Myocardial recovery was more evident in women, leading to significantly better transplantation-free survival rates, with women averaging 96% at 4 years vs. 84% for men (*p* = 0.03) [4].

**Figure 2:**
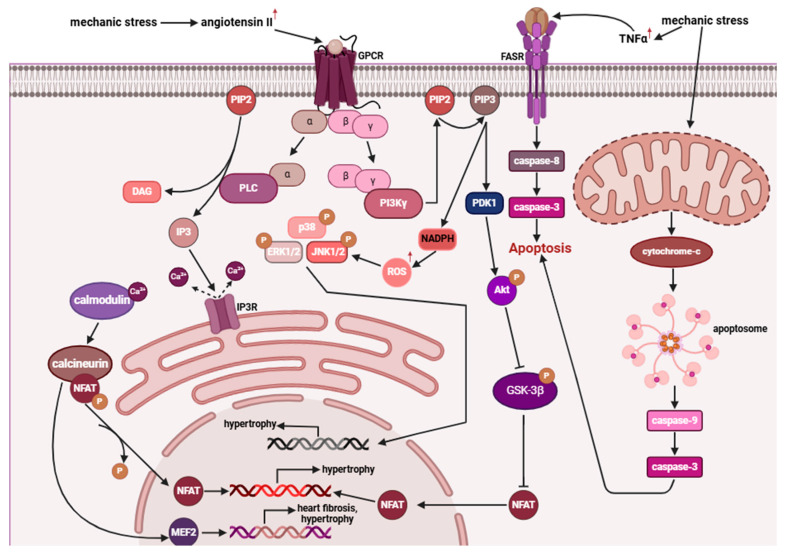
Potential molecular pathway for dilated cardiomyopathy development. Increasing mechanical stress leads to elevated levels of angiotensin II, which stimulates the G-protein-coupled receptor. The α-subunit of this receptor activates phospholipase C, which breaks down PIP2 into IP3. IP3 stimulates the release of calcium into the cytoplasm. When Ca^2+^ binds to calmodulin, calcineurin is activated, which in turn, activates the transcription factors MEF2 and NFAT. The β and γ subunits of the GPCR also activate signaling pathways through the activation of PI3Kγ, which converts PIP2 into PIP3. This leads to the activation of substrates required for the formation of PDK1, which phosphorylates Akt and thereby inhibits GSK-3β. As a result, NFAT is not deactivated by phosphorylation and is transported into the nucleus, enhancing cardiac hypertrophy. The previously formed PIP3 also activates NADPH oxidase, contributing to increased levels of ROS and activation of the MAPK pathway through phosphorylation of the p38, ERK1/2, and JNK1/2 subunits, which stimulate the expression of genes responsible for hypertrophy. Mechanical stress can also lead to cell death by increasing TNFα levels, which, through interaction with the FAS receptor, activates Caspase 8, as well as through mitochondrial membrane damage and apoptosome activation. Created in BioRender. Rintz, E. (2025) https://BioRender.com/bhc02ja.

Characteristic symptoms of DCM include arrhythmias, atrial or ventricular, and sudden cardiac death. Other symptoms of DCM are those of congestive heart failure, like dyspnea, congestive edema, edema, orthopnea. It is reported that characteristic manifestations also include thromboembolic events [8]. Most of them start between 20 and 60 years of age, but children can be affected with DCM constituting up to 60% of childhood cardiomyopathies. In familial and idiopathic DCM, patients can be asymptomatic for a long time or even lifetime, while signs of inherited DCM like chamber dilation, reduced ejection fraction, or fibrosis, could have been detected at an earlier stage [2]. However, phenotype is not limited to pathologies within the heart muscle, as the mutated genes associated with DCM development can encode proteins that are also fundamental components of other muscles in the body as well. DCM often appears as a “symptom disease,” arising because of other conditions, such as myofibrillar myopathies and muscular dystrophies, whose symptoms are typically associated with the degeneration of skeletal muscles [11,58]. Additionally, in patients with DCM, progressive muscle wasting can be observed, occurring as a result of inflammation caused by heart failure [59]. However, the phenotype of DCM is dependent on a complex molecular mechanism which begins with the onset of mechanical stress, caused by the impaired function of the heart muscle, which results from the deformation and dysfunction of cardiomyocytes after the mutations. This leads to a significant increase in the level of angiotensin II, which, by binding to the G-protein-coupled receptor (GPCR), activates G protein subunits [60]. One of these subunits activates PI3kγ, which phosphorylates PIP2 to PIP3, leading to the activation of NADPH, increasing the level of reactive oxygen species (ROS). Cellular stress activates MAPK pathway by stimulating kinase cascade (Figure 2) [60,61]. Furthermore, PIP3 activates the pathway where PDK1 phosphorylates Akt, which inhibits GSK-3β. Therefore, there is an excessive proliferation of cardiomyocytes [60]. At the same time, the other subunit of the G protein activates phospholipase C, which cleaves PIP2 into IP3 and DAG. IP3 binds to the IP3 receptor on the sarcoplasmic reticulum, resulting in excessive release of Ca^2+^ into the cytoplasm. The binding of calcium to calmodulin activates calcineurin, which dephosphorylates NFAT, allowing it to translocate to the nucleus, thereby activating genes associated with heart fibrosis, cardiac remodeling, and reduced contractile efficiency [60,62,63]. As mentioned earlier, mechanical stress can also contribute to the activation of apoptotic pathways, as it leads to an increase in TNF-α levels, which, by activating the FAS receptor, triggers the activation of caspases 8 and 3. Stress can also directly damage mitochondria, resulting in the release of cytochrome c, formation of the apoptosome, and the activation of caspase-9 and caspase-3 [60,62].

Since the development of the disease is the result of multiple factors, many diagnostic tests are performed. As a first step, a family history analysis is performed along with a physical examination to evaluate the patient’s general condition. Laboratory tests, such as a complete blood count and assessments of kidney and liver function, are also performed [2]. Imaging studies, particularly echocardiography and cardiac magnetic resonance imaging, are crucial for identifying characteristic anomalies such as heart chamber morphology and heart muscle tissue characterization. In cases where there is a positive family history, genetic testing is conducted to detect specific mutations. It is important to rule out other potential conditions, such as coronary artery disease or other types of cardiomyopathies as well [2]. Since DCM is often a cause of heart failure (HF), treatment reflects the management of chronic HF. The proposed treatment includes angiotensin-converting enzyme inhibitors or angiotensin II receptor blockers (ARB) for patients with ACEI intolerance, each associated with beta-blockers. For still symptomatic patients, mineralocorticoid receptor antagonist (MRA) should be considered in addition to ACEI and beta-blocker. ACEI, ARB, beta-blockers, and MRA have demonstrated to reduce the risk of hospitalization and death in patients with heart failure and reduced ejection fraction. For patients with idiopathic DCM, a disease-modifying therapy (including ACEI, beta-blocker, MRA, ivabradine and/or cardiac resynchronization therapy) may show substantial or even complete recovery of LV systolic function [64]. Additionally, exercise therapies have been noted to improve cardiopulmonary function and increase the quality of life in cardiac patients, although there is limited data regarding this type of therapy in patients with DCM and the exercise prescription varies in frequency, intensity, time and type. Based on a systematic review, the exercise frequency ranged from three to five times a week and exercise intensity were prescribed within a range from 50% to 80%. The exercise time was as high as 45 min by the final month of prescription, and the exercise type was mainly aerobic exercise and resistance training [65]. For non-ischemic DCM, cardiac rehabilitation improved cardiopulmonary outcomes, compared to patients without rehabilitation, not causing any adverse effects [65]. Since the most common cause of DCM is mutations, several gene therapy approaches are also distinguished. Some of these involve silencing mutated genes, for example using RNAi with ASP-RNA (mutations in the *TNNT2* gene) and siRNA (mutations in the *LMNA* gene associated with Hutchinson-Gilford progeria syndrome), as well as antisense oligonucleotides regulating mRNA transcription (mutations in the *TTN* and *PLN* genes) [66,67,68,69]. Gene editing tools, such as CRISPR, are also used to remove gene fragments (*TTN* gene) and TALEN, which allows for the introduction of corrections in the sequence (R14del mutation of the *PLN* gene) [70,71]. Another approach involves using viral vectors, such as AAV, which can carry not only the corrected version of the mutated gene but also genes whose products can support the affected organ (*XINB, VEGF-B*, and *S16EPLN* genes that improve heart function and have protective effects) [72,73,74]. In DCM, there are also mutations involving a premature stop codon, preventing the production of a complete protein. In one such case, a drug was used to help produce the full product (*PTC124* and the *MLNA* gene) [75,76]. Autophagy stimulation should also be considered as a therapeutic target, despite dilated cardiomyopathy not being typically classified as a storage disease, because impairment of this process has been observed in individuals with DCM, including mTOR1 activation, accumulation of ubiquitinated proteins and p62, as well as the presence of aggresomes [77]. Interestingly, another study found that autophagy is associated with positive left ventricular remodeling in patients with DCM, suggesting that stimulating this process could be very significant. Enhancing autophagy may help reduce the risk of complications, provide additional energy for the heart, and support its structural remodeling [78]. Natural autophagy stimulators that may alleviate DCM-related pathologies include resveratrol, which activates the process through the AMPK pathway; 1,25-Dihydroxyvitamin D3, which influences the pathway by inhibiting mTOR; and metformin [79,80,81]. Additionally, caloric restriction has also been observed to stimulate autophagy [82].

The field of precision medicine presents clear potential in the treatment of DCM, but there are limitations to the knowledge base which is a roadblock in the yield of genetic testing. Therefore, improving the yield of genetic testing, and identification of high-risk genotypes, elucidating impact of disease-modifying factors and deep phenotyping should be focused on as future research targets to improve treatment of DCM through precision medicine [83]. Since current treatments for DCM use medications and devices that are generally applied in heart disorders, future research should focus on more specialized approaches targeting the causes and effects of DCM. It is also crucial to distinguish between the genetically driven development of the disease and external factors, as the current understanding is uncertain [84]. A key area for future research is the in-depth exploration of molecular pathways, from mutations to the progression of heart pathology, in order to better understand the course of the disease. Such insights could help in selecting appropriate therapies and medications [84]. It is important to focus on discovering future targets of therapy and developing those that already exist. As an example, upregulation of fractalkine/CX3CR1 together with CMV seropositivity and consequent migration of immune and inflammatory cells can be associated with worse outcomes in patients with inflammatory related DCM. Thus, further studies are required to identify a subgroup of at-risk patients with DCM, which in turn, can influence future drug trials utilizing immune modulators to target that pathway. Genes *BEX1*, *RGCC*, and *VSIG4*, and the HIF-1 signaling pathway can be mentioned as other targets for future treatment, as they have been identified using integrated bioinformatics analysis to play a crucial role in pathology of DCM [85].

### 2.2. Metabolic Myopathies

Metabolic myopathies are a heterogeneous group of disorders caused by enzymatic defects in cellular energy metabolism, specifically involving the catabolism of carbohydrates, fatty acids, or mitochondrial oxidative phosphorylation. These disorders predominantly affect skeletal muscles but may also involve other systems depending on the specific metabolic pathway affected [31]. They are broadly categorized based on the affected metabolic pathway, with the main groups being glycogen storage diseases (GSDs), fatty acid oxidation (FAO) defects, and mitochondrial myopathies.

Clinically, metabolic myopathies manifest through a wide spectrum of symptoms, which can be divided into static presentations, such as progressive muscle weakness, and dynamic presentations, including acute, recurrent symptoms triggered by exertion or metabolic stress. Progressive muscle weakness is characteristic of conditions like GSD II (Pompe disease), GSD III (Cori disease), and GSD IV (Andersen disease), while dynamic symptoms, such as post-exercise muscle pain, cramps, or rhabdomyolysis are frequently observed in GSD V (McArdle disease), FAO defects, and mitochondrial myopathies [31,86]. In regions where baseball is popular, the “home run” sign—difficulty sprinting around bases due to exercise-induced muscle spasms—characterizes young patients with McArdle disease. Conversely, prolonged exercise, like hiking or soccer, causing myalgias, fatigue, and pigmenturia without acute contractures, suggests FAO defects. For instance, patients with CPT II deficiency may experience fatigue and pigmenturia during fasting or prolonged activities, such as military training marches. Patients with mitochondrial diseases often experience exercise intolerance, marked by premature fatigue disproportionate to weakness, alongside exercise-induced or fixed manifestations [86].

Symptom severity and age of onset vary widely. In neonates and infants, metabolic myopathies are often present as hypotonia, feeding difficulties, or multisystemic involvement, including cardiac or respiratory failure. In contrast, older children and adults more commonly exhibit exercise intolerance, recurrent myoglobinuria, or progressive muscle weakness. External stressors, such as infections, fasting, or vigorous exercise, can precipitate life-threatening complications, including metabolic crises, rhabdomyolysis, and organ failure [87]. For instance, untreated infantile-onset Pompe disease historically had >90% mortality by age one, but enzyme replacement therapy (ERT) has improved 5-year survival to 83%. Late-onset forms (e.g., McArdle disease, CPT II deficiency) typically manifest in adolescence or adulthood, with near-normal lifespans if managed appropriately [31]. Mitochondrial disorders, like MELAS syndrome, have a median survival of 17 years post-symptom onset, often due to stroke-like episodes or cardiac complications [88].

Although metabolic myopathies are rare or ultra-rare diseases, their collective prevalence is not insignificant, accounting for approximately 2% of cases referred for suspected myopathy [87]. Among GSDs, the overall incidence ranges from 1 in 20,000 to 43,000 live births, with GSD V affecting approximately 1 in 100,000 individuals and GSD II affecting 1 in 20,000 to 43,000 individuals, though Pompe disease is also classified as a lysosomal storage disorder due to glycogen accumulation within lysosomes caused by acid alpha-glucosidase (GAA) deficiency [89]. FAO defects show considerable variability in prevalence by subtype; for example, multiple acyl-CoA dehydrogenase deficiency (MADD) occurs in 1 in 15,000 to 20,000 births in the United States, while primary carnitine deficiency affects 1 in 30,000 to 142,000 individuals [90,91]. Mitochondrial myopathies, the most common among these disorders, have an estimated prevalence of 1 in 4300 individuals, with MELAS incidence estimated at 1 in 8500 births [88,92].

Accurate and timely diagnosis is essential to optimize management and prevent complications. A comprehensive evaluation includes a detailed medical and family history, neurological examination, and laboratory tests, such as creatine kinase levels, blood acylcarnitine profiles, and urine organic acid analysis. Advanced diagnostic tools, like electromyography, nerve conduction studies, muscle biopsies, and genetic testing, particularly next-generation sequencing, have revolutionized the identification of metabolic myopathies [93,94]. Early diagnosis facilitates targeted therapies, such as enzyme replacement therapy in GSD II or dietary interventions in FAO defects, which can significantly improve patient outcomes [93]. Metabolic myopathies encompass approximately 30 distinct conditions, though the exact number may vary depending on the classification system and ongoing advancements in genetic research. In this review, I will focus on a subset of these disorders, particularly those that contribute to skeletal muscle degradation [86].

Glycogen storage diseases are a group of inherited metabolic disorders caused by enzymatic defects that result in abnormalities in glycogen metabolism. Among them, Pompe disease, Cori disease, and McArdle disease differ in pathomechanism, clinical manifestation, and therapeutic approach, although some symptoms, such as myopathy, may overlap. GSDs can be present at various ages, ranging from the neonatal period to adulthood [95,96,97].

GSD II is caused by mutations in the *GAA* gene, leading to lysosomal deficiency of acid alpha-glucosidase and pathological accumulation of glycogen within lysosomes, which disrupts organelle function and cellular homeostasis [97,98]. This glycogen buildup triggers lysosomal enlargement and excessive V-ATPase activity, destabilizing lysosomal pH gradients and stabilizing mammalian target of rapamycin complex 1 (mTORC1) interaction with the lysosomal membrane via Ragulator-Rag GTPase complexes, even during starvation. Consequently, mTORC1 remains constitutively anchored to lysosomes, impairing its inactivation and sustaining ULK1 phosphorylation, which blocks autophagy initiation causing a reduced level of LC3-II/LC3 ratio. Concurrently, energy deficits from defective glycogen digestion activate the AMPK-TSC2 pathway, which inhibits Rheb, yet mTORC1 retains partial activity due to its proximity to lysosome-bound Rheb, exacerbating autophagic failure and accumulation of damaged organelles [99,100]. These defects critically limit ERT, where recombinant human GAA (rhGAA) relies on mannose-6-phosphate (M6P) receptor-mediated lysosomal targeting. To address these limitations, strategies, like glycoengineering rhGAA to enhance M6P residues, suppressing autophagy (via Atg5/7 inactivation), or activating mTOR signaling (via TSC2 knockdown), have shown promise in improving ERT efficacy by redirecting resources toward glycogen clearance [101,102,103,104]. Complementary approaches, including autophagy-modulating compounds and gene therapy, e.g., AAV-mediated GAA delivery, aim to restore lysosomal function and mitigate secondary pathologies, though immune responses and sustained expression remain challenges [105]. Early diagnosis via newborn screening is vital to initiate ERT before irreversible muscle damage occurs [97].

In GSD III, a defect in the glycogen debranching enzyme (AGL) caused by *AGL* gene mutations leads to the accumulation of structurally abnormal glycogen in the cytosol of cells [106]. Treatment primarily involves dietary interventions, including frequent meals rich in complex carbohydrates and cornstarch [95]. Currently, no enzyme replacement therapy exists for Cori disease. Rehabilitation plays a crucial role in maintaining physical fitness, and regular moderate-intensity exercise can improve muscle strength while avoiding intense physical activity [95].

GSD V, caused by mutations in the *PYGM* gene, results in a deficiency in muscle glycogen phosphorylase, impairing glycogen utilization as an energy source during exercise [107]. Treatment focuses on physical activity and dietary adjustments. Moderate exercise is recommended, with avoidance of sudden, intense exertion. Pre-exercise carbohydrate consumption is advised to provide quick energy [108]. The characteristic “second wind” phenomenon in McArdle disease refers to improved exercise tolerance after a few minutes of activity as the body shifts to alternative energy sources [109]. Research is underway to evaluate creatine supplementation and a diet rich in carbohydrates. Rehabilitation involves regular, moderate-intensity exercise tailored to individual capabilities, avoiding strenuous activity. Notably, unlike Pompe disease, glycolysis in McArdle disease is not completely impaired, as muscle fibers can still convert glucose to glucose-6-phosphate (G6P). No enzyme replacement therapy exists for GSD V [110,111].

The diagnosis of GSDs involves biochemical tests, including measuring serum creatine kinase (CK) levels, which are typically elevated in GSD V and GSD II; exercise tests, such as the ischemic forearm test or cycle test to detect abnormal lactate production in GSD V; histopathological muscle analysis showing increased glycogen accumulation (PAS staining) and/or absence of specific enzyme activity (e.g., muscle phosphorylase in GSD V); genetic testing to confirm mutations in *GAA* (GSD II), *AGL* (GSD III), or *PYGM* (GSD V); newborn screening to identify GSD II early; and enzymatic assays measuring enzyme activity in muscle biopsies or blood samples when genetic results are inconclusive [31,97].

Fatty acid oxidation defects, including disorders, such as multiple acyl-CoA dehydrogenase deficiency and primary carnitine deficiency (PCD), are characterized by an autosomal recessive inheritance pattern and belong to the spectrum of lipid storage myopathies. However, in CPT II deficiency, lipid accumulation in skeletal muscles is not observed [31]. A common feature of these disorders is impaired fatty acid metabolism, which leads to lipid accumulation in skeletal muscles and clinical manifestations, such as muscle weakness, exercise intolerance, myalgia, and episodes of rhabdomyolysis with myoglobinuria. Triggers, such as fever, fasting, prolonged physical activity, emotional stress, and cold exposure, can exacerbate symptoms. MADD syndrome is characterized clinically by strokes that are atypical in that they usually occur in people less than 40 years old and generally do not conform to large vessel distribution [112].

The FAO metabolic pathway begins with the transport of long-chain fatty acids and very long-chain fatty acids (LCFAs, VLCFAs) into mitochondria via the acylcarnitine transporter and the enzymes carnitine palmitoyltransferase I (CPT I) and II (CPT II) [113]. Within the mitochondria, fatty acids undergo β-oxidation, an essential process for energy production in conjunction with the electron transport chain and oxidative phosphorylation. In PCD, mutations in the *SLC22A5* gene, encoding the carnitine transporter, disrupt carnitine transport into muscle cells, thereby impairing fatty acid transport into the mitochondria and leading to an energy deficit [31]. Reduced levels of carnitine in plasma and tissues are caused by approximately 150 pathogenic variants identified in the *SLC22A5* gene [31,113,114]. In MADD, mutations in the *ETFA*, *ETFB*, and *ETFDH* genes result in defects in acyl-CoA dehydrogenases, which are critical for β-oxidation. Notably, mutations in *ETFDH* are frequently associated with the late-onset form of the disease and may lead to secondary coenzyme Q10 deficiency [115].

Diagnosis of these disorders relies on genetic analysis and biochemical tests. In PCD, histological examination of muscle tissue reveals the presence of vacuoles and lipid accumulation, predominantly in type I fibers. CK levels can vary widely, ranging from normal to up to 15 times the reference values. In MADD, muscle histology may show lipid droplet accumulation, COX-negative fibers, and ragged red fibers (RRFs) [116]. Clinically, MADD presents in three major forms: a neonatal-onset form with congenital anomalies, a neonatal-onset form without anomalies, and a milder or late-onset form that often responds to riboflavin supplementation [115,116].

Treatment strategies for MADD and PCD involve dietary modifications, avoidance of prolonged fasting, and, in some cases, supplementation with carnitine, riboflavin, and coenzyme Q10 [115,116]. Avoidance of symptom-triggering factors is also crucial. Genetic studies, including next-generation sequencing (NGS), play a vital role in the precise diagnosis of these conditions and the identification of novel genetic variants [31].

In these disorders, autophagy is not the primary compensatory mechanism; however, cellular stress caused by metabolic disturbances can influence it. Animal models with M/SCHAD deficiency suggest that autophagy plays a role in adapting to metabolic stress [117]. Modulating the mTOR pathway and activating TFEB can stimulate autophagy, indicating potential therapeutic approaches through the removal of toxic metabolites.

Future therapies include gene therapies aimed at repairing genetic defects and chaperone drugs that stabilize misfolded enzymatic proteins. Mouse models with gene knockouts, such as the M/SCHAD deficiency model, are used to analyze pathomechanisms and test new strategies, such as treatment with triheptanoin, which may serve as an alternative energy source [113,118]. Pharmacological chaperones work by stabilizing misfolded enzymatic proteins resulting from genetic mutations. These compounds bind to the defective enzyme, helping it achieve proper spatial conformation, thereby restoring its catalytic function [119].

Mitochondrial myopathies are a diverse group of rare genetic disorders in which mitochondrial dysfunction leads to impaired cellular energy production, particularly in muscle cells. These disorders present a wide spectrum of clinical manifestations, ranging from mild exercise intolerance to severe, multisystem dysfunctions, including myopathies. The complexity of these conditions arises from the variety of genes encoding mitochondrial proteins, located in both the nuclear and mitochondrial genomes, resulting in a broad range of phenotypes and pathophysiological mechanisms [86].

Primary mitochondrial myopathies encompass various disorders caused by mutations in over 1200 genes responsible for mitochondrial functions [119]. Among these, mitochondrial encephalopathy with lactic acidosis and stroke-like episodes (MELAS), Kearns–Sayre syndrome, and myoclonic epilepsy with ragged red fibers (MERRF) are notable examples, each with distinct clinical features. Exercise intolerance is a common symptom in mitochondrial diseases, often presenting as premature fatigue even during mild activities, such as climbing a single flight of stairs. After resting briefly, patients can typically resume activity, but symptoms quickly return [120]. Unlike those with glycogen storage disorders, these patients rarely experience stiffness, cramps, or the second wind phenomenon [121]. They frequently report muscle heaviness or burning during exertion, with exercise intolerance often being more pronounced than muscle weakness. This symptom may occur alone or alongside muscle weakness and multisystemic involvement [86].

The pathophysiology of mitochondrial myopathies centers on impaired respiratory chain function, leading to ATP deficiency and excessive production of ROS [122]. This results in damage to muscle cells and other tissues, such as the brain, heart, nerves, and internal organs. Depending on the mutation’s location and its effect on mitochondrial function, clinical symptoms can vary significantly [123].

MELAS is characterized by stroke-like episodes, often resulting in permanent neurological deficits, headaches, vomiting, and seizures. Other symptoms include lactic acidosis, myopathy, exercise intolerance, muscle weakness, and, in some cases, cardiac complications. The condition is most associated with mutations in the mitochondrial tRNA (Leu)(UUR) gene (m.3243A>G), which disrupt the synthesis of mitochondrial tRNA proteins and consequently impair respiratory chain function. The severity of the disease and the age of symptom onset vary, depending on the level of mutation heteroplasmy. This mitochondrial DNA (mtDNA) damage, primarily inherited maternally, can be transmitted to next generations [123].

Kearns–Sayre syndrome, another example of mitochondrial myopathy, presents progressive external ophthalmoplegia, pigmentary retinopathy, heart block, and ataxia. Its pathogenesis involves large-scale deletions in mtDNA, leading to respiratory chain complex dysfunction and energy deficits [123,124].

In diagnosing mitochondrial myopathies, histopathological examination of muscle tissue may reveal ragged red fibers, mitochondrial abnormalities, and lipid accumulation. Muscle biopsy remains a crucial diagnostic tool, particularly when DNA testing does not confirm a diagnosis [123].

Treatment approaches for mitochondrial myopathies include symptom management, vitamin and cofactor supplementation, dietary modifications, and aerobic exercise. In MELAS, arginine is used to improve endothelial function and reduce stroke-like episodes. Additionally, supplementation with vitamins, L-carnitine, and coenzyme Q10 supports respiratory chain function [122]. Managing rhabdomyolysis and avoiding triggers are essential to prevent complications. Deoxynucleoside therapy (e.g., deoxycytidine and deoxythymidine) has shown promise in restoring mitochondrial DNA levels and improving muscle strength and respiratory function in TK2-deficient patients. This approach aims to bypass the enzymatic defect and provide substrates for mitochondrial DNA synthesis [108]. Also compounds like rapamycin (an mTOR inhibitor) and resveratrol (a sirtuin activator) have been explored for their ability to enhance autophagy in mitochondrial disorders. Rapamycin inhibits mTORC1, a key regulator of autophagy, while resveratrol activates SIRT1, promoting mitochondrial biogenesis and autophagy [120]. These approaches are particularly relevant in conditions like MELAS and Kearns–Sayre syndrome, where mitochondrial dysfunction is central to disease pathology [120].

**Figure 3:**
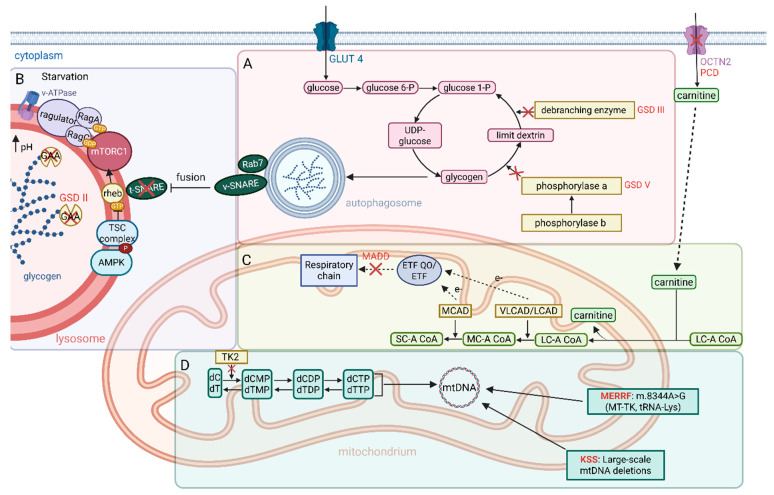
(**A**) Schematic representation of glycogen metabolism in muscle cells including selected enzymatic defects in GSD. Glucose supplied by the GLUT transporter 4, is phosphorylated in the cytoplasm to form Glucose-6-phosphate (glucose 6-P) and then converted to glucose-1-phosphate (glucose 1-P). Glucose 1-P serves to synthesize UDP-glucose, which is the direct donor of glucose residues in glycogenesis. UDP-glucose is incorporated into the structure of glycogen, which is the spare form of glucose in muscle. In the process of glycogenolysis, glycogen is degraded by glycogen phosphorylase (phosphorylase a and its inactive form, phosphorylase b), leading to the formation of dextrins; the lack of glycogen phosphorylase leads to GSD V. Dextrins are then branched leading to the formation of glucose 1-P by debanching enzyme; its absence will result in GSD III. (**B**) In response to increased pH levels in the lysosome due to glycogen accumulation, V-ATPase activity increases. V-ATPase is crucial for maintaining the Ragulator-Rags complex on lysosomes, which in turn bound to GTP attracts and localizes mTORC1 to the surface of lysosomes. Once mTORC1 is located, Rheb-GTP activates the kinase. This causes mTORC1 to be attached to lysosomes during both starvation and nutrient sufficiency. It does not function properly in either situation—during starvation, mTOR is not fully activated. Despite the presence of inhibitors (AMPK and TSC2 complex) on lysosomes, the continuous proximity of Rheb counteracts full mTOR deactivation. This results in lysosome distortion and dysfunction of the t-SNARE protein, preventing proper fusion of the autophagosome with the lysosome and delivery of glycogen to the lysosome. (**C**) Schematic analysis of mitochondrial fatty acid β-oxidation. Long-chain, medium-chain, and short-chain acyl-CoA (LC-A CoA, MC-A CoA, SC-A CoA) are oxidized by the respective dehydrogenases: VLCAD/LCAD (very/long-chain acyl-CoA dehydrogenase) and MCAD. (**D**) Mitochondrial DNA (mtDNA) metabolism and effects of specific mutations. Created in BioRender. Rintz, E. (2025) https://BioRender.com/519h7hq.

In conclusion, metabolic myopathies represent a diverse group of inherited disorders caused by defects in cellular energy metabolism, affecting pathways such as glycogenolysis, fatty acid oxidation, and mitochondrial oxidative phosphorylation (Figure 3). These disorders manifest through a spectrum of clinical presentations, ranging from progressive muscle weakness to dynamic symptoms triggered by exertion or metabolic stress, with significant variability in age of onset and severity [113]. Advances in diagnostic tools, including genetic testing and muscle biopsy, have enhanced the identification of these conditions, enabling targeted therapies such as enzyme replacement, dietary modifications, and exercise regimens [125,126]. Despite their rarity, metabolic myopathies collectively impact a notable proportion of individuals, underscoring the importance of early diagnosis and intervention to mitigate complications and improve outcomes. Ongoing research into novel treatments, including gene therapy and autophagy modulation, holds promise to address the underlying pathophysiology and expanding therapeutic options [120]. Ultimately, a multidisciplinary approach is essential for optimizing the management of these complex disorders and improving the quality of life for affected individuals.

### 2.3. Myofibrilal Myopathies

Myofibrillar myopathies (MFMs) are very rare diseases characterized by dissolution of myofibril and progressive accumulation of Z-disk proteins in aggregates. These diseases in most cases can be inherited by an autosomal dominant approach or sporadic appearance [127,128]. Characteristic MFM phenotype includes abnormal aggregation not only of Z-disk proteins but also proteins that interact with them. Various mutations in different genes may cause an MFM phenotype. Previously there were only six genes in which mutation was known for causing MFM. These genes consist of *DES*, *CRAYB*, *MYOT*, *LDB3/ZASP*, *FLNC*, and *BAG3*. However, recently ten additional genes in which mutation was responsible for MFM-like phenotype were observed in patients [11,127]. These newly identified mutations are yet to be understood in terms of mechanism and pathophysiology, thus are referred as “MFM-like”. Each gene with a mutation responsible for MFM disease makes its specific type.

MFM1 is caused by a mutation in the *DES* gene, with the most common being a single nucleotide substitution for proline, which disrupts protein correct conformation; also, not only SNP, but deletions on *DES* gene could cause MFM1 as well, with many possible SNP and deletion sites [129]. Pathomechanism of MFM1 may differ depending on the mutation site. Desmin is a scaffold protein for many components, thus, a mutation in different sites may result in a wide range of phenotypes, such as proteasome inhibition or even mitochondria abnormalities [130,131,132]. *CRYAB* gene encodes a protein called Alpha-crystalin B chain (HspB5), which is part of the HSPB family. In skeletal muscles, its function is to prevent misfolding of proteins, inhibition of apoptosis, and modulation of membrane fluidity. Mutation in HspB5 results in MFM2, with characteristic aggregates of mutant protein and desmin resulting in progressive muscle weakness; however, MFM2 commonly comes with cataract, which is an unusual symptom in MFM disorders, specific for MFM2 [133,134]. Mutation in *MYOT* causes MFM3; this type is defined by the accumulation of products coming from myofibril disintegration with characteristic myofibrillar myopathy symptoms, as this protein plays an important role in sarcomere assembly, interacting with filamin C and a-actinin [135]. Another MFM type is caused by a mutation in the *LDB3* gene with a single nucleotide substitution in the actin binding site; however, binding to actin is not disrupted. The protein is responsible for z-disc integrity during muscle contraction. Mutant protein causes aggregation of FLNC and Hsp-70, thus stalling removal of damaged and misfolded FLNC, which is what leads to muscle fiber disintegration. Binding affinity of mutant protein is not reduced; therefore, this proves that MFM4 phenotype does not come from ligand binding disruption. Mutant LDB3 interacts indirectly with BAG3 protein that scaffolds chaperone-assisted selective autophagy (CASA) complex, through Hsp-70 that binds with FLNC as well as LDB3. This interaction disrupts autophagy, but the late onset of MFM4 suggests that the disturbance of CASA is not as severe as in MFM6 caused by *BAG3* mutation or is compensated by other protein control mechanism, due to its indirect interaction [136]. MFM5 is caused by mutations in the *FLNC* gene, with onset between the fourth and sixth decade. FLNc interacts with various z-disc proteins and also cross-links actin filaments. Aggregates observed in MFM5 consist not only of FLNc but also of CASA complex proteins, thus reducing efficiency of autophagy [137]. All MFM types are related to aggregates formation, either by interaction with CASA complex, being part of this complex, or by entrapment of certain CASA agents. Therefore, we would like to showcase to a further extent, MFM6 pathophysiology and its mechanism, as this certain type is caused by a mutation in Bag3, that is a scaffold protein of CASA complex, which plays a main role in aggregate clearance and often interacts with proteins responsible for MFM disease, thus linking all MFMs.

**Figure 4:**
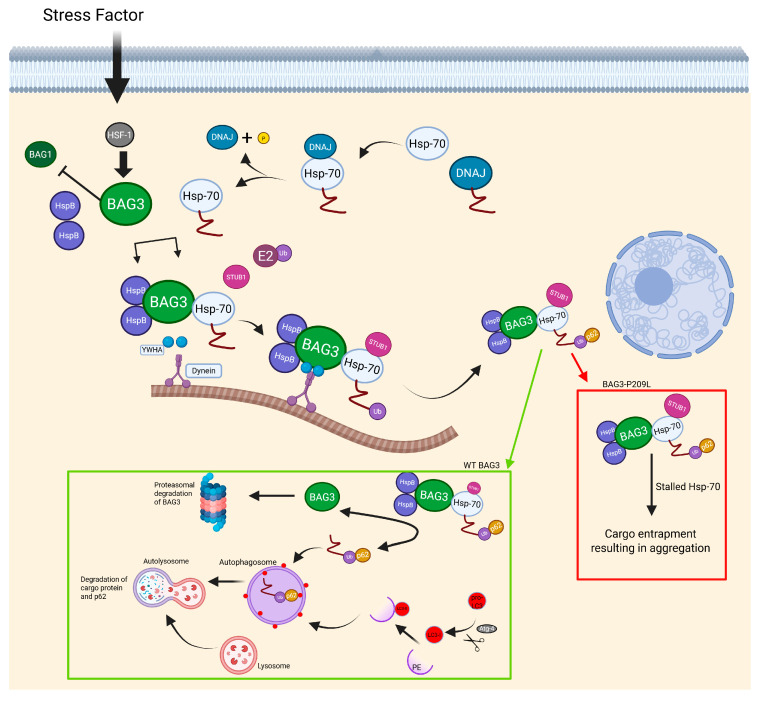
During stress conditions, cells prioritize degradation by autophagy rather than proteasomes. BAG3, by having a higher affinity for Hsp-70 than BAG1, creates a complex with heat shock proteins and cargo proteins guided for degradation. A protein designated for degradation is previously ubiquitinated and binds with p62 complex with WT BAG3 releasing cargo protein upon contact with LC3-II, thus creating autophagosome which reacts with lysosome making autolysosome; meanwhile, BAG3 is guided for proteasomal degradation after successful cargo guidance. Cells presenting P209L BAG3 mutation cannot release cargo protein, because of Hsp-70s toxic gain of function in this complex, resulting in whole complex entrapment and formation of aggregates [138,143,152,153,154,155,156]. Created in BioRender. Rintz, E. (2025) https://BioRender.com/ahsr8iq.

BAG3 is a multifunctional protein, responsible for keeping proteostasis in CASA complex, as well as inhibiting apoptosis. Given its multifunctionality, there are five identified binding sites: WW binding with proline-rich ligands, two separate IPV motives, which bind with small heat shock proteins, especially with HspB8, PXXP site that makes it possible for CASA complex to bind with dynein, and a fifth motive is called BAG, which allows for binding to Hsp70 [132,138]. The most common mutation responsible for MFM6 is a single nucleotide substitution from proline to leucine at the 209th nucleotide of the *BAG3* gene. However, different mutations responsible for MFM6 were reported, such as substitution from proline to glutamine, also at 209th nucleotide, or substitution from proline to serine at the 470th nucleotide. However, both unusual mutations were responsible for way less severe symptoms in patients [139,140]. A mutation on the 209th nucleotide colocalizes with a second IPV motive. Even though mutation occurs in the IPV motive, the Bag3 still maintains the ability to bind with HspB8, even more so, its binding effectiveness stays close to normal; therefore, this indicates that the MFM6 phenotype does not come from the disruption of HspB8 binding [141]. Rather, a possible explanation of the MFM phenotype resulting from a P209L mutation is impairing of the chaperon-associated autophagy by stalling the Hsp-70 protein (Figure 4), described as a toxic gain of function [141]. This so-called toxic gain of function results in the formation of aggregates including CASA complex proteins as well as selected degradation components. Therefore, this further reduces the number of active autophagy proteins because of their entrapment in aggregates [142,143]. The formation of toxic MFM-related aggregates comes from a chaperone-assisted selective autophagy due to its complex insufficiency of releasing a ubiquitinylated client (Figure 4) [143]. Second, a widely observed symptom of MFM diseases is disintegration of muscle fibers, caused by deficient degradation of damaged FLNc protein, which undergoes unfolding and refolding during muscle contraction. Bag3 is responsible for targeting damaged FLNc, which is why its reduced presence causes the accumulation of damaged FLNc and, therefore, muscle disintegration [142]. This muscle disintegration further leads to many symptoms with progressive character.

Such symptoms are rigid spine, scoliosis, joint contractures, and elevated CK levels. Also, it is well known that MFM comes with muscle weakness or even muscle atrophy. MFM6 is a highly progressive disorder, which with time, may even result in cardiomyopathy or respiratory failure [11,144]. What also differs from MFM6 is the fact that most MFMs onset around adulthood, while thodr caused by a P209L mutation onset in childhood with early lethality due to cardiomyopathy or respiratory complications [145]. All of these symptoms observed in patients may only be seen when a single allele holds a P209L mutation. Mice exhibiting homozygous mutations present early lethality, just a few days after birth [146]. Even though MFMs appear with certain well-known phenotype, proper diagnosis requires advanced diagnostic tools. This can start with a muscle biopsy in order to observe abnormalities such as shape irregularity of muscle fibers, size differences, centrally located cells nuclei, and earlier, well described protein aggregates. However, differences between types of MFMs, especially with the same onsets, forces usage of additive diagnostic tools in order to establish a diagnosis. One of such techniques is immunostaining, which allows us to see protein aggregates, thanks to the antibodies specific to certain proteins [11]. A technique that is also worth taking into consideration in terms of diagnosis is immunoblotting. It has been proven that some MFMs, compared to WT muscle cells, present higher band intensity in an immunoblot assay, related to proteins potentially causing disease [147]. These methods allow for the recognition of Myofibrillar myopathy; however, to find genes accountable for such phenotype, genetic analysis must be provided. Genome analysis allows for the recognition of mutation and, therefore, is the final step in the diagnosis of MFM disorders [11].

To this date, there is no therapy, nor treatment that would provide cure or improve phenotype of patients with MFM6 as well as other MFM diseases [11]. Although, researchers started to wonder if inducing autophagy may result in improvement of aggregate clearance in MFM caused by P209L mutation. Scientists examined 71 compounds of which only 13% showed a reduction in aggregates [148]. All of the 71 tested stimulators induced autophagy, but in a different manner as well as in different concentrations; that is why only few drugs showed improved clearance. Out of these nine compounds, there were two that significantly performed better than the others. Metformin and amiodarone, with amiodarone being neurotoxic and metformin being a safer option, thus putting metformin as a potential autophagy drug for MFM6 [148]. Metformin, a wide range of signaling pathways, is promising in MFM research; however, it still requires optimalization and additional experiments. Yet another interesting possibility of future therapy for MFMs is gene editing by CRISPR-CAS9. Edition with specific mechanisms like CRISPR-CAS9 allows for the inactivation of a mutated allele by nonsense-mediated decay or prevention of transcription, thus reducing the amount of mutant protein [149]. Unfortunately, this method is far from being implemented, because of the complexity and risk that comes from using this technique in vivo, including the safety and efficiency of delivering CRISPR-CAS9 components into human cells. Yet another significant difficulty that comes with this method is potential off-target activity [150,151].

To sum everything up, MFM diseases have yet to be understood. To this day, there are certain aspects that remain unknown, the same way potential therapies are yet to be discovered. However, there are mechanisms that showcase improvement of MFM phenotype in muscle cells, thus providing opportunity for future studies, with autophagy induction in broad light. Current research and studies mostly focus on the development of new autophagy stimulants which could help with aggregate clearance, but also genetic modification that could interfere with a mutated allele. In addition to that, there are also studies which center around the improvement of known beneficial factors such as the CRISPR-CAS9 complex or known autophagy inducing drugs.

### 2.4. Congenital Myopathies

Congenital myopathies (CMs) are a heterogenous group of rare diseases. The group consists of genetic muscle disorders with symptoms appearing throughout the lifespan. Patients with these diseases show dysmorphic facial features and muscle weakness [157]. Prevalence of CMs estimates 1.62 (96% CL 1.13–2.11) per 100,000 [158]. CMs are divided by histopathological symptoms, including the presence of nemaline rods, centralized nuclei, core lesions within muscle fibers, fiber-type disproportion and cytoplasmic inclusions. Nemaline myopathy is characterized by rod-like inclusions in muscle [157]. The prevalence of this disease is estimated to be 0.20 per 100 000 (95%Cl: 0.10–0.35) in the general population and 0.22 per 100,000 (95% Cl: 0.03–0.40) in the pediatric population [158]. Centronuclear myopathies occur in two types of inheritance: X-linked form and autosomal form. The prevalence in the general population is estimated at 0.08 per 100,000 in the general population and 0.44 per 100,000 in the pediatric population [158]. This group is characterized by specific organization of nuclei and/or internalized nuclei [159]. Central core disease (CCD) and minicore disease (MmD) are caused by a mutation in different genes, but both reveal themselves as cores in muscle fibers. The condition affects approximately 0.37 per 100,000 in the general population and 0.46 per 100,000 in the pediatric population [158]. While CCD is caused by a mutation in the *RYR1* gene and revealed as single, usually centrally located core at the full length of muscle fiber, MmD is caused by a mutation in the *SEPN1* gene with multiple small cores spread along the muscle fiber [159]. Congenital fiber type disproportion (CFTD) is visible as the hypertrophy of type 1 muscle fibers compared to type 2 with the absence of structural abnormalities [159]. The estimated prevalence in the general population is 0.23 per 100,000 (95% CI: 0.04–0.42) and 0.25 per 100,000 (95% Cl: −0.05–0.54) in the pediatric population [158]. Myopathies with cytoplasmic inclusions are caused by a mutation in a rod domain of the *MYH7* gene; they appear as a myosin storage in the type 1 fibers [159]. It has been reported that these diseases are associated with a variety of genes and their differential expressions. Sarcomeric gene mutations further disrupt thin filament assembly and function, as seen in NEB which encodes nebulin, a large structural protein crucial for proper thin filament length regulation and stabilization (mutations manifest as lack of C-terminal SH3 domain) [160], TPM affect tropomyosin, a key regulatory protein that controls actin–myosin interactions by modulating calcium sensitivity during muscle concretion (mutations present as aberrant isoforms with altered Ca^2+^ activity) [89,161]. Abnormal protein turnover contributes to the regulation of nebulin ubiquitination via Kelch family proteins—KLHL40 and KLHL41 [162,163]. KLHL40 binds nebulin fragments and leimodin-3 (LMOD3), inhibits K48-linked polyubiquitination, and thus prevents proteasome-mediated degradation of these substrates, ensuring proper thin-filament assembly [162], KLHL41 interacts directly with nebulin’s super-repeat regions and the nebulin-related anchoring protein (NRAP) is itself polyubiquitinated and acts as a molecular chaperone to prevent aggregation and degradation of nebulin and NRAP, thereby maintaining sarcomere integrity [163], while KBTBD13 interacts with Cullin-3 (CUL3) in proteasomal pathways. KBTBD13 is a member of the Kelch-BTB protein family; its BTB domain binds the scaffold protein CUL3, while its Kelch repeats recognize specific substrates. CUL3 is a core scaffold of a multisubunit E3 ubiquitin ligase that attaches ubiquitin chains to target proteins. KBTBD13 acts as a BTB-Kelch substrate adaptor for the CUL3 E3 ubiquitin ligase complex; it recruits specific sarcomeric proteins for ubiquitination and proteasomal degradation, thereby regulating muscle fiber protein turnover [164]. Membrane trafficking defects arise from *DNM2* mutations that impair T-tubule maintenance and BIN1-DNM2 interactions, leading to BIN1 overexpression and tubular aggregate formation [165,166]. Oxidative stress and calcium dysregulation occur in SEPN1-related myopathy through selenoprotein N deficiency, reduced sarcomeric reticulum (SR) Ca^2+^ load, and caffeine-insensitive RyR1 channels [167]. CACNA1S variants affecting Cav1.1 accelerate calcium release defects, and TTN splice defects in the PEVK region destabilize the thick filament. These final pathways, aberrant calcium release and thick-filament destabilization, represent key convergence points for contractile failure in several CM. Mutations in CACNA1S, which encodes the Cav1.1 L-type calcium channel implicated in disorders, such as hypokalemic periodic paralysis and malignant hyperthermia susceptibility, disrupt excitation–contraction coupling and impair SR Ca^2+^ release [168]. Despite diverse genetic causes, these pathways converge on impaired sarcomere integrity, dysregulated Ca^2+^ homeostasis, and defective autophagy-proteasome function.

MTM1-associated myopathy (also known as X-linked myotubular myopathy) is a rare disorder caused by a mutation in the *MTM1* gene. The *MTM1* gene encodes myotubularin, a lipid phosphate which is involved in membrane trafficking, phosphoinositide metabolism, and endosomal homeostasis in muscle cells. Myotubularin is also responsible for dephosphorylation of phosphatidylinositol 3-phosphate (PI3P) [169]. Mutations in this gene lead to the loss or dysfunction of myotubularin. In physiological conditions, myotubularin regulates PI3P levels to ensure proper membrane trafficking and autophagosome formation. In pathophysiological conditions, dysregulation of myotubularin activity leads to pathological accumulation of PI3P in muscle cells. An increase in PI3P level causes disruption of autophagy by dysregulated recruitment of PI3P-binding proteins, which are necessary for autophagosome formation [170]. In consequence, autophagosomes are either improperly formed or fail to mature, which leads to the accumulation of damaged proteins and dysfunctional organelles inside the muscle fibers. In addition, the persistence of cellular debris and dysfunctional organelles triggers chronic cellular stress, exacerbates sarcomeric disorganization, and promotes progressive muscle fiber atrophy [171,172]. Chronic impairment of autophagy contributes to cellular stress, muscle fiber atrophy, and muscle weakness. Thus, dysregulated PI3P metabolism represents a key molecular mechanism that connects MTM1 mutations with defective autophagy and muscle weakness observed in affected patients.

There are several therapeutic approaches to congenital myopathies, which have been recently investigated. A strategy that has demonstrated potential involves the modulation of PI3P metabolism. Inhibition of the PI3P-producing kinase PIK3C2B decreased pathological PI3P accumulation and improved muscle structure and function [171]. Another approach focuses on modulation of the activin receptor type IIB (ActRIIB) pathway. ActRIIB inhibition has been found to promote muscle hypertrophy by stimulation of satellite cell proliferation and activation of the Akt/mTOR signaling pathway. Mtm1 p.R69C knock-in mice, which model the human phenotype of X-linked myotubular myopathy, were treated with ActRIIB-Fc. ActRIIB-Fc is a soluble, engineered fusion protein that mimics the extracellular domain of the ActRIIB receptor, functioning similarly to monoclonal antibodies by binding to and blocking ligands, such as myostatin, form interacting with their native receptor. This strategy is based on modulation of cell activity and activation of anabolic pathways, notably Akt/mTOR, resulting in muscle regeneration and growth. ActRIIB-Fc blocks the inhibitory signals regulating muscle growth, particularly those mediated by myostatin and other TGF-β family ligands. This treatment resulted in significant muscle fiber hypertrophy, improved muscle contractility, and an extended lifespan compared to untreated controls. In summary, ActRIIB inhibition enhances satellite cell activity and promotes anabolic signaling pathways, such as Akt-mTOR, leading to muscle regeneration and growth [173,174]. Another strategy includes gene therapy. Recent clinical trials are based on AAV-mediated delivery of a functional copy of the *MTM1* gene. One of the conducted trials, AT132, utilizes an AAV8 vector carrying the human *MTM1* gene under the control of a muscle-specific promoter. In preclinical models, this includes XLMTM mice and dogs. XLMTM mouse model is the *Mtm1* knockout (*Mtm1*-/y) mouse, in which the *MTM1* gene is completely inactivated. These mice exhibit muscle hypertrophy, fiber atrophy, centralized nuclei, defective T-tubule organization, and reduced lifespan. AT132—a gene therapy strategy based on recombinant AAV serotype 8 (rAAAV8), which delivers a functional copy of the *MTM1* gene under a muscle-specific promoter-restored myotubularin-1 production, normalized phosphoinositide metabolism, corrected T-tubule abnormalities, and significantly improved muscle strength and survival [171,172]. Furthermore, gene therapy is increasingly explored as a therapeutic option for other forms of congenital myopathies. One of the therapeutic strategies is the rAAV-shDnm2, which lowers DNM2 production, rescuing myofiber architecture in Dnm2-knockout models of centronuclear myopathy. rAAV-shDnm2 is a gene therapy approach that uses a recombinant adeno-associated virus to deliver small hairpin RNA (shRNA) targeting the *DNM2* gene. This strategy is designed to lower the expression of DNM2 protein. By reducing the levels of DNM2, the therapy aims to rescue the architecture of myofibers and improve muscle function in models of disease. The shRNA binds to the mRNA of the *DNM2* gene, leading to its degradation and thereby inhibiting the production of the mutant protein [175]. Therapy based on AAV9-shRNA usage, which modulates ORAI1 production, downregulates the Orai1 Ca^2+^ entry channel. That results in partially restored calcium homeostasis and muscle function in STIM1-related myopathy models [176].

CMs are a diverse group of genetic muscle disorders characterized by disruptions in sarcomere structure, membrane trafficking, calcium homeostasis, and protein degradation pathways. Among them, X-linked myotubular myopathy is caused by *MTM1* mutations, which lead to pathological PI3P accumulation, impaired autophagy, and muscle atrophy. Recent therapeutic advances, including PI3P metabolism modulation, ActRIIB pathway inhibition, and AAV-mediated gene therapy, such as AT132, may become promising strategies for the treatment.

### 2.5. Distal Myopathies

Distal myopathies are characterized by progressive muscle weakness and atrophy beginning in the distal parts of the limbs. These diseases can manifest from early childhood to late adulthood. Distal myopathies are genetically heterogeneous; at this point, there are 25 different genes known to be responsible for their occurrence and 20 forms of the disease differing in clinical presentation, age of muscle involvement, and histopathological criteria. Despite their considerable diversity in terms of clinical manifestations and genetic determinants, they belong to the group of muscular dystrophies—genetic conditions characterized by the progressive loss of muscle fibers [177].

Among the many forms of distal myopathies, Welander distal myopathy, Udd myopathy (tibial muscular dystrophy), Nonaka myopathy (GNE myopathy), Markesbery-Griggs distal myopathy (MFM4), Miyoshi myopathy type 2 (MMD2), and Laing myopathy exemplify how specific genetic mutations contribute to divergent clinical manifestations. Notably, MFM4 is also classified within the group of myofibrillar myopathies, emphasizing the molecular and pathological overlap between distal myopathies and other broader categories of muscle disease. This reinforces the notion that myopathies, despite clinical distinctions, often share common mechanistic features. Each of these disorders demonstrates a unique molecular defect that leads to distinct histopathological features, progression rates, and patterns of muscle involvement. For instance, Welander myopathy involves mutations in the *TIA1* gene, leading to early hand muscle weakness and rimmed vacuoles [178]. Histopathological changes in the muscle include muscle fiber damage, fiber degeneration, fibrosis, and rimmed vacuoles, which are intracellular inclusions containing membrane bodies, glycogen, and amorphous material. Immunohistochemistry of muscle biopsies from WDM patients showed strong staining for TIA1, TDP43, G3BP, and p62, which colocalize in areas adjacent to fringed vacuoles to form aggregates. Under conditions of cellular stress, TIA1 promotes the accumulation of messenger ribonucleoprotein complexes (mRNPs) in stress granules, leading to the inhibition of translation [178]. The first symptom is most often weakness of the thumb and/or index finger with clumsiness in small precision movements which spreads to the other fingers and results in an inability to extend the fingers. Gradually, a weakness of the distal parts of the lower extremities develops. The most-affected muscle is the anterior tibial muscle, leading to the inability to raise the toes and difficulty in standing on the heels, which results in walking difficulties and steppage gait [178].

Distal UDD myopathy results from titin gene mutations, with symptoms predominantly affecting foot dorsiflexors and a generally slower disease course. The effect of the mutation is to disrupt the structure and function of the tittin protein, leading to progressive muscle weakness. One of the main functions of titin is to keep the contractile elements of the sarcomere in place and is responsible for muscle elasticity. Titin also provides multiple ligand-binding sites for a large number of other muscle proteins. including the muscle-specific protease calpain-3, α-actinin, myosin, myomysin, and telethonin [179]. Serum creatine kinase levels are normal or slightly elevated in comparison to healthy patients, and electromyography (EMG) shows myopathic features. Muscle biopsy shows dystrophic tissue with muscle fibers having single and multiple vacuoles. The incidence of AD TMD in Finland is between 5 and 15 cases per 100,000 people. Symptoms of muscle weakness usually appear after the age of 40 and initially affect the dorsal flexor muscles of the foot. In non-Scandinavian patients, weakness may gradually involve the extensor muscles of the fingers and wrists and, over time, the proximal muscles. In most Finnish patients, the disease progresses more slowly, rarely affecting the upper limb muscles or proximal muscles [180].

**Figure 5:**
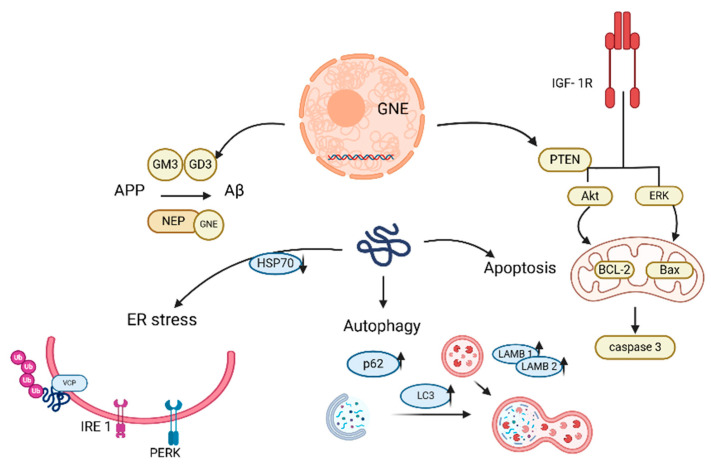
Molecular events present in the GNE myopathy. Abnormal GM3 and GD3 lead to Aβ synthesis disorder in the Golgi apparatus and endoplasmic reticulum of GNE. At the same time, hyposialylated NEP cannot clear Aβ. Aβ deposition generates ER stress in GNE-mutant cells, which further triggers survival or apoptotic signaling mediated by IRE1-α or PERK, respectively. Molecular chaperones, ERAD and UPS, are all involved in the clearance of misfolded proteins. In the case of Aβ deposition, the autophagy–lysosome pathway is activated immediately to correct or degrade Aβ. Many molecules related to apoptosis in GNE myopathy, such as caspase 3 and IGF-1R, control cell survival and apoptosis by regulating the balance between AKT and ERK. Increased presence of autophagy markers, such as p62, LC3, and lysosome membrane-associated proteins (LAMP1 and LAMP2), were detected in patients’ muscle biopsies. Created in BioRender. Rintz, E. (2025) https://BioRender.com/8v71k21.

In contrast, Nonaka myopathy—also known as distal myopathy with rimmed vacuoles (DMRV) or GNE myopathy—is caused by mutations in the *GNE* gene, which encodes a bifunctional enzyme with UDP-GlcNAc2-epimerase and ManNAc kinase activities. These mutations disrupt sialic acid metabolism, leading to broader systemic effects, including autophagic dysfunction and protein aggregation. GNE catalyzes the rate-limiting step in the sialic acid biosynthesis and MNK catalyzes the next step (Figure 5) [181]. Muscle biopsies of affected muscles show variation in muscle fiber size, the presence of characteristic “rimmed” vacuoles, and typically, a lack of inflammation [182]. Ultrastructural and immunohistochemical findings in GNE myopathy strongly suggested that autophagy and apoptosis are important contributors to myofibrillar degeneration, marginal vacuolization, and muscle fiber atrophy [183]. Reports indicated that mutations in *GNE* disrupt mitochondrial structure and function, leading to cell apoptosis [184]. Autophagy is impaired at the degradation and clearance stage of the cell, leading to pathological accumulation of autophagic vacuoles and protein deposits [185]. In addition, cells with GNE myopathy show a characteristic Aβ (amyloid beta) accumulation [186]. However, diagnosis is still based on symptoms of muscle weakness with early onset of foot drop, biopsy of affected muscles, and genetic testing [187]. Bosch-Morató et al. proposed a disease mechanism in which amyloidosis may result from hypozialylation, ultimately causing apoptosis of muscle fibers in GNE myopathy [188]. Aβ oligomers have been shown to directly enter the membrane and form a porous structure called “amyloid channels” that are selectively permeable to Ca^2+^, leading to a rapid increase in Ca^2+^ levels and disrupting Ca^2+^ homeostasis in cells [189]. Symptoms of the disease mainly appear in the 3rd decade of life and begin with distal weakness of the forelegs, most commonly the tibia, accompanied by foot drop. A common phenotypic feature is the preservation of the quadriceps muscle until late in the disease process. The distribution of weakness in the upper limbs can be variable, with the intrinsic muscles of the hand and the deep flexors of the fingers being most affected. In general, respiratory function is not significantly affected in patients [190]. The prevalence of GNE myopathy is estimated at ~1 to 9/1,000,000 [190].

The study indicates altered calcium homeostasis in GNE mutant cells that may affect the autophagy phenomenon observed in these cells. Calcium chelator may be useful in restoring normal calcium levels in GNE mutant cells to restore cellular functions affected by altered calcium [191]. Other studies suggest therapeutic options, such as modulators of the HSP70 chaperone (BGP-15), cofilin activator (CGA), and ligands, such as IGF-1 (Insulin-like Growth Factor Receptor), which may help rescue cellular defects caused by GNE dysfunction [183,192,193]. Dysregulation of HSP70 with increased expression levels of BAG3, JNK, and BAX was observed in GNE mutant cells. Downregulation of HSP70 in cells with mutations in the *GNE* gene suggests a disruption in normal protein folding, which affects the JNK signaling pathway. This pathway is important for the cellular response to stress, and its dysregulation can lead to increased oxidative stress, apoptosis, or other cellular disorders [192]. BGP-15, an activator of HSP70, reduced phenotypic defects resulting from GNE mutations, such as protein aggregation, and improved cell function [192].

Abnormal sialylation can lead to dysfunction in actin signaling, leading to abnormal cell migration; Cofilin can significantly reverse the cell migration defect [193]. In GNE-deficient cells, the IGF-1R receptor is hyposialylated. Hyposialylation of IGF-1R reduced the efficiency of signaling following IGF-1 binding. Activation of this receptor by IGF-1 ligand may protect cells from apoptosis. This mechanism could be used to develop therapies for diseases associated with mutations in the *GNE* gene [183]. Acenouramic acid (Ace-ER) and ManNAc have been investigated as drugs to slow the progression of GNE myopathy. Ace-ER progressed from preclinical studies to the completion of phase 3 clinical trials in 2017. Results from preclinical studies and early clinical trials indicated stabilization and a slower rate of muscle function deterioration. The phase 3 study did not reach any primary or secondary endpoints and, therefore, concluded that Ace-ER was safe and had no or a very little effect on the progression of GNE myopathy [194]. The autophagic flux in GNE myopathy is impaired as indicated by elevated levels of p62 and LC3II. Research shows that metmorphine may be a potential treatment for patients with GNE myopathy by restoring autophagic flux and through the AMPK/mTOR-independent pathway [195].

In 2010, four doses of *GNE* gene lipoplex were administered intramuscularly to the patient, improving muscle function, *GNE* gene expression, and sialic acid, with no serious side effects. The GNE-wt-DNA vector, created from human GNE cDNA and the pUMVC3 expression vector, was used for lipoplex delivery. In 2011, seven doses were administered intramuscularly in a follow-up study, achieving stabilization of muscle strength and an increase in sialic acid-related proteins. Despite promising results, the therapy was not further developed or clinically implemented [196].

Another distal myopathy is Miyoshi early adult-onset type 2 (MMD2) that is caused by a mutation of the dysferlin gene on chromosome 2p13. Dysferlin is a membrane protein in the sarcolemma and is involved in various functions such as membrane repair and vesicle fusion, T-tubule development and maintenance, Ca^2+^ signaling, and regulation of various molecules [197,198]. Patients’ monocytes have increased phagocytosis activity. Serum creatine kinase levels significantly increased 10-fold to 150-fold from the upper limit of normal, also in preclinical stages. The MRI pattern of patients’ muscles and their biopsies shows various fibers sizes, necrotic and regenerative fibers, and fat and connective tissue accumulation [199]. The age of onset for MMD2 is 20 ± 5 years, respectively, and patients present calf or thigh weakness and atrophy. In the following 5 years, weakness and atrophy evolved to include the upper limbs [200]. Most patients present with lower limb weakness, which is proximal, distal, or both for patients. An upper limb weakness is also reported by 7% of patients. Their symptoms include muscle wasting, pain, stiffness, or cramps, or pseudohypertrophy [201]. A clinical trial currently evaluating the safety, efficacy, and tolerability of SRP-6004, is a recombinant AAVrh74 gene therapy expressing a dysferlin transgene under the control of a muscle-specific promoter (rAAVrh74.MHCK7.DYSF.DV), which is administered intravenously to ambulatory patients [NCT02710500] (clinicaltrials.gov).

A common feature of distal myopathies are mutations in the genes encoding muscle proteins resulting in the dysfunction of these proteins and disruption of the biochemical cellular pathways in which they participate [180]. All types of distal myopathies are characterized by a slowly progressive loss of muscle strength in the distal parts of the body. Their diagnosis is based on similar diagnostic tests, such as muscle biopsy, genetic testing, electromyography, creatine kinase levels. Regardless of the specific mutation, a common feature of distal myopathies is the accumulation of damaged proteins and organelles and apoptosis of muscle cells, leading to progressive muscle weakness and atrophy [9].

These diseases differ mainly in their genetic background, the specificity of their clinical manifestations, such as age of onset, time of progression, location of the affected muscles, blood creatine kinase levels, and muscle biopsy results. No definite diagnosis can be made on other grounds than identification of the final molecular genetic defect. Besides usual investigations, including EMG and muscle biopsy, muscle imaging is very important in defining the precise pattern of muscle involvement; this is based on the combination of age at onset, mode of inheritance, pathology, and muscle imaging, and the number of underlying candidate genes. The diagnosis of distal myopathies can be complicated because the symptoms may be non-specific and similar to other conditions. In clinical practice, distal limb weakness of myopathic origin is rare and, therefore, other neuromuscular disorders must be considered, including motor neuron disease and polyneuropathies [202].

### 2.6. Idiopathic Inflammatory Myopathies

Idiopathic inflammatory myopathies (IIMs) are a group of autoimmune conditions involving muscle inflammation (myositis) and weakness. This umbrella term comprises a few types and subtypes into which IIMs are classified (Table 1) as per their clinical manifestations [203]. IMMs share the clinical manifestation of progressive immune-mediated muscle injury. General symptoms (excluding IBM) span subacute symmetrical proximal pattern of muscle weakness, associated myalgia and muscle tenderness, bulbar/esophageal striated muscle weakness (difficulties swallowing) and respiratory muscle weakness (nocturnal hypoventilation). Extra muscular symptoms associated with IIMs may include interstitial lung disease (ILD), cardiac abnormalities (such as prolonged corrected QT interval (QTc), conduction blocks, atrial or ventricular ectopic beats, systolic dysfunction, left ventricular diastolic dysfunction, hyperkinetic left ventricular contraction, myocardial inflammation, degenerative changes, and necrosis), and possible underlying malignancies [204,205]. Cancer diagnosis can either precede, coincide with, or follow the diagnosis of IIMs. The risk of cancer is highest during the first year after the diagnosis of IIMs, suggesting that IIMs may develop in a paraneoplastic process [206,207]. However, more specific symptoms need to be analyzed for a differential diagnosis. DM, IMNM/NAM, and OM display similarly, with proximal weakness and higher CK level, whereas IBM involves long finger flexors and quadriceps and shows no or only moderate rise in CK (Table 1).

The key tools in diagnosing IIMs include serological testing, magnetic resonance imaging, muscle biopsies, and electromyography. Further distinguishing into subtypes is possible based mainly on MSA (myositis-specific antibodies), histopathological analysis, and CK level measurements, which also serves as an indicator of treatment response, making it a valuable biomarker. On top of that, taking detailed family history is advised, with special attention to possible muscular dystrophy or proximal motor neuropathies [203].

The epidemiology of IIMs is understudied, especially with South America, Africa, and Asia remaining unreported. However, IIMs are generally considered rare diseases with the prevalence of 2–25 per 100,000 [208]. Some studies report figures for certain populations. Amongst these, the prevalence of IIMs was found to be 13.9 per 100,000 [209]. In Oman, the prevalence rates for DM, PM, and JDM were 2.2, 2.2, and 1.14 per 100,000 population, respectively, with the overall mortality rate determined as 6.9% (this fraction of patients passed away during the study period of 16 years, from 2006 to 2022). This study found that the 5-year survival rates were high (94.4% for PM, 91.7% for DM, and 89% for JDM) but declined significantly over a 10-year period (67%, 69%, and 83.3%, respectively), indicating disease progression and complications over time. The average age of patients in the study was 38.78 years, with juvenile onset cases (21 out of 116 patients) occurring as young as under 12 years old [210]. A Swedish study reported a mortality rate of 60 per 1000 person-years in IIM and 20 per 1000 person-years in the general population [211].

Even though much is understood about the pathological mechanisms leading to the development of IIMs, the exact cause remains unknown. The clinical and histopathological discrepancies between the subtypes suggest that specific processes underlying each of them are different; however, the overall mechanism could be generalized to the following description. The development of IIMs may be triggered by viral/bacterial infections, trauma, or genetic predisposition separately, but a synergistic combination of genetic, environmental, and immunological factors could also lead to the development of IIMs [212,213]. Injuries or UV radiation directly damage muscle cells. As a result, damage associated with molecular patterns (DAMPs) is released. DAMPs bind to toll-like receptors (TLRs) activating immune cells. Thereby, the NF-κB (nuclear factor kappa-light-chain-enhancer of activated B cells) pathway, which controls DNA transcription, cytokine production, and cell survival, is activated. Through a cascade of events, NF-κB enters the nucleus and upregulates proinflammatory genes, which stimulates cytokine and chemokine production, perpetuating and intensifying further inflammation. On the other hand, infections or genetic susceptibility provoke changes in skeletal muscles, such as abnormally high expression of Major Histocompatibility Complex 1 (MHC-1). Hence, they begin to present autoantigens (fragments of their own cleaved proteins) to CD8+ T lymphocytes by which they are recognized as foreign. This induces CD8+ T cells to infiltrate muscle cells with a high expression of MHC-1, atypical for muscle cells (CD8–MHC-1 complex is characteristic of PM and IBM). Consequently, they release perforin granules and granzyme B, ultimately resulting in myonecrosis [214]. Furthermore, many proteins (e.g., FHL1) are substrates for granzyme B. The gene encoding four-and-a-half LIM domain 1 (FHL1) is expressed predominantly in skeletal muscles, and its function primarily involves muscle growth, differentiation, and structural maintenance. Some patients develop autoimmunity to FHL1 (granzyme B released by cytotoxic cells cleaves FHL1, and cleaved FHL1 fragments become neo-epitopes, likely initiating the development of autoantibodies), which later leads to muscle fiber damage [215]. Then, the sequence of events is as above, starting with the release of DAMPs, leading up to cytokine and chemokine production, coupled with further expression of MHC-1 and antigen presentation to helper T cells. Mutations that have been reported as possible contributors to the pathogenesis of IIMs mainly affect genes responsible for proper muscle structure and function, e.g., mutations in the PDZ domain of the *LDB3*/*ZASP* gene (in the example of IBM). ZASP (Z-band alternatively spliced PDZ-motif protein), also known as LDB3 (LIM domain binding 3), is a sarcomeric PDZ-LIM protein expressed in skeletal and cardiac muscle at Z-disc. ZASP is important for the stabilization of the sarcomeric structure and the PDZ domain is predicted to be involved in the Z-line integrity of skeletal muscle [136,147,216].

A clear consensus regarding the safety and effectiveness of physical therapy in patients with IIMs has not been reached yet [217]. Nevertheless, several studies show evidence of the potential benefits of rehabilitation. In patients with PM and DM, aerobic exercise and resistance training on moderate-high intensity were found to promote aerobic capacity and alleviate muscle impairment and activity limitations [218]. Evaluations of specific long-duration training plans have also been reported—patients assessed after a 24-week program combining a supervised training of activities of daily living (ADL), resistance, muscle strengthening, and stability showed prevented progressive deterioration along with clinically meaningful improvement [219].

Corticosteroids are the first-line treatment for the majority of patients with IIM as they help quickly establish disease control. However, further immunosuppression is required to prevent disease recurrence upon steroid discontinuation. Mild cases may be effectively controlled by incorporating steroid sparing agents, such as azathioprine, mycophenolate, or methotrexate [203]. A retrospective review of medical records found that IVIG (intravenous immunoglobulin) dosed at 2 g/kg monthly within the first 3 months to adult patients with NAM diagnosis was associated with a bigger likelihood of attaining marked improvement or returning to baseline at 6 months. However, this difference was not sustained at follow-up, which could be because of the too small sample (63 patients analysed) [220]. For treatment-resistant DM, IVIG also showed promising results. In a double-blind, placebo-controlled study of 15 patients (18–55 years old), eight patients assigned to 2 g/kg of IVIG per month for 3 months had a significant improvement in scores of muscle strength (*p* < 0.018) and neuromuscular symptoms (*p* < 0.035) [221]. The results acquired from the recent double-blind, randomized, placebo-controlled, multicenter, phase 3 study confirm the safety of administering IVIG in adult patients with DM, but emphasize the need for monitoring for thromboembolic events (TEEs) [222]. For refractory IIMs, rituximab (RTX) has been reported to be effective. A retrospective analysis after six months of 26 patients treated with RTX (1000 mg intravenously, twice, with a 2-week interval) found a significant reduction in CK levels (*p* = 0.001) compared to the baseline, improved muscular strength (*p* < 0.001), and a reduction in the extramuscular activity of the disease (*p* < 0.001). RTX led to improvements particularly in DM skin rash, arthritis, and pulmonary symptoms. Additionally, this treatment enabled a reduction in the mean daily dose of steroids needed to control disease activity (*p* = 0.002) [223]. Belimumab treatment (10 mg/kg) tested in a 40-week multicenter, randomized, double-blind, placebo-controlled trial showed no statistically significant differences between arms [224]. Similarly, there was no significant difference between tocilizumab and placebo arms in the 24-week multicenter, randomized, double-blind, placebo-controlled trial in refractory adult patients with DM and PM (8 mg/kg intravenously/placebo every 4 weeks) [225].

Ectopic expression of local immune modulators could limit inflammation more effectively than systemic immunosuppressive treatment. A clinical trial investigated the delivery of the human follistatin gene (huFS344) via recombinant AAV serotype 1 (rAAV1) to quadriceps muscles of both legs in patients with sporadic IBM. Results of the study demonstrated ameliorations in performance, as evidenced by increased distances in the 6-min walk test (median +56.0 m/year for treated subjects, compared to −25.8 m/year (*p* = 0.01) in untreated subjects), along with histological improvements, such as reduced fibrosis and increased muscle fiber size. While these findings indicate that follistatin gene therapy may promote muscle regeneration and help counteract atrophy in moderately affected, ambulatory patients, treating individuals in advanced disease stages with more severe muscle loss poses challenges [226]. Another strategy for genetic treatment of IIMs may focus on fostering the repair or allowing for the replacement of damaged muscle. Myotrophic factors, including utrophin or insulin-like growth factors, could promote muscle regeneration. Furthermore, the use of stem cells or myoblasts might provide therapeutic value. Myoblasts are precursor muscle cells that can form new muscle fibers by fusion or integration into existing ones. Thus, transplantation of healthy donors or autologous myoblasts can help repopulate muscle tissue. Though clinical evidence is still limited, a recent study showed that transplanting one million GFP-labeled myoblasts mixed with Matrigel into the tibialis anterior muscle of mice led to the generation of over 1000 new myofibers and an increase in muscle weight by approximately 10% [227]. Transplantation of genetically corrected myoblasts with the aim of restoring normal protein expression should be investigated as a potential therapeutic strategy because very limited research has been carried out so far.

The biggest need for future studies seems to be a more detailed differentiation between specific subtypes of IIMs, including the mechanisms of pathogenesis. That would provide much more thorough ground for a better identification of therapy targets and novel treatments. Tissue engineering could serve as a powerful tool for creating IIMs’ disease models. In terms of therapies, there is a need to examine the use of stem cells and myoblasts. Stem cell therapy includes three promising aspects—immunomodulation, regeneration of damaged muscle, and tissue protection. Immunosuppressive properties of mesenchymal stem cells (MSCs), such as inhibition of T-cell proliferation via production of IL-10 and reduction in IFN-γ, could be advantageous in IIMs’ treatment [228,229]. On the other hand, muscle-derived stem cells or induced pluripotent stem cells (iPSCs) can differentiate into muscle fibers and hence help replenish damaged muscle tissues, which could tackle muscle atrophy and fibrosis associated with chronic inflammation [230,231]. In addition, exosomes and growth factors (e.g., IGF-1, VEGF) released by stem cells help protect existing muscle tissue, reduce scarring (fibrosis), and enhance vascularization. Therapies based on adenoviral vectors may raise difficulties, as persistent transgene expression remains a challenge (these vectors tend to exist episomally within the nucleus, which leads to transient expression) [232]. Furthermore, efforts are needed to prevent a viral immune response from being induced. Strategies that have been explored include immunosuppressive agents and vector modifications (minimizing the expression of immunogenic viral proteins by removing all viral genes, as in helper-dependent adenoviral vectors) [233,234]. Research is also advisable to achieve high transduction efficiency in all muscles, not only regenerating ones [235].

### 2.7. Dystrophies

Muscular dystrophies are a group of rare genetic disorders of a wide spectrum. They can be inherited or appear de novo. The substance of this disease is the splitting of muscle fibers, resulting in their atrophy and replacement by connective and fatty tissue. Early onset of the disease is associated with a more severe phenotype, resulting in reduced life expectancy [236,237,238]. Due to heterogeneity of approaches conducted in studies, there is no accurate epidemiological data of muscular dystrophies. Several groups of muscular dystrophies have been distinguished based on their clinical presentation and pathogenesis. These include Duchenne muscular dystrophy, Becker muscular dystrophy, Emery-Dreifuss muscular dystrophy, myotonic dystrophy, facioscapulohumeral dystrophy, limb girdle muscular dystrophy, congenital muscular dystrophies, and distal muscular dystrophies. The prevalence of Duchenne muscular dystrophy (DMD) was estimated at 4.8 per 100,000 people, while the prevalence of Becker muscular dystrophy (BMD) was estimated at 1.6 per 100,000 people. For myotonic dystrophy, facioscapulohumeral dystrophy (FSHD), Emery-Dreifuss muscular dystrophy (EDMD), and limb girdle muscular dystrophy, the prevalence per 100,000 people was estimated at 8.3, 4.0, 0.4, and 1.6, respectively [237,239]. Data suggests that myotonic dystrophy type 2 appears less frequently in the population than myotonic dystrophy type 1. However, there are no established prevalence estimates [240]. According to studies, life expectancy for DMD has increased over time from 25 to 32 years with a median survival rate of 28 years. This is most likely due to the fact that the quality of patient care has improved, as well as the introduction of steroid medication as standard treatment, cardiac medication, the alleviation of disease symptoms, which is associated with an increase in patient independence, and lastly, mechanical ventilation [238,241].

**Figure 6:**
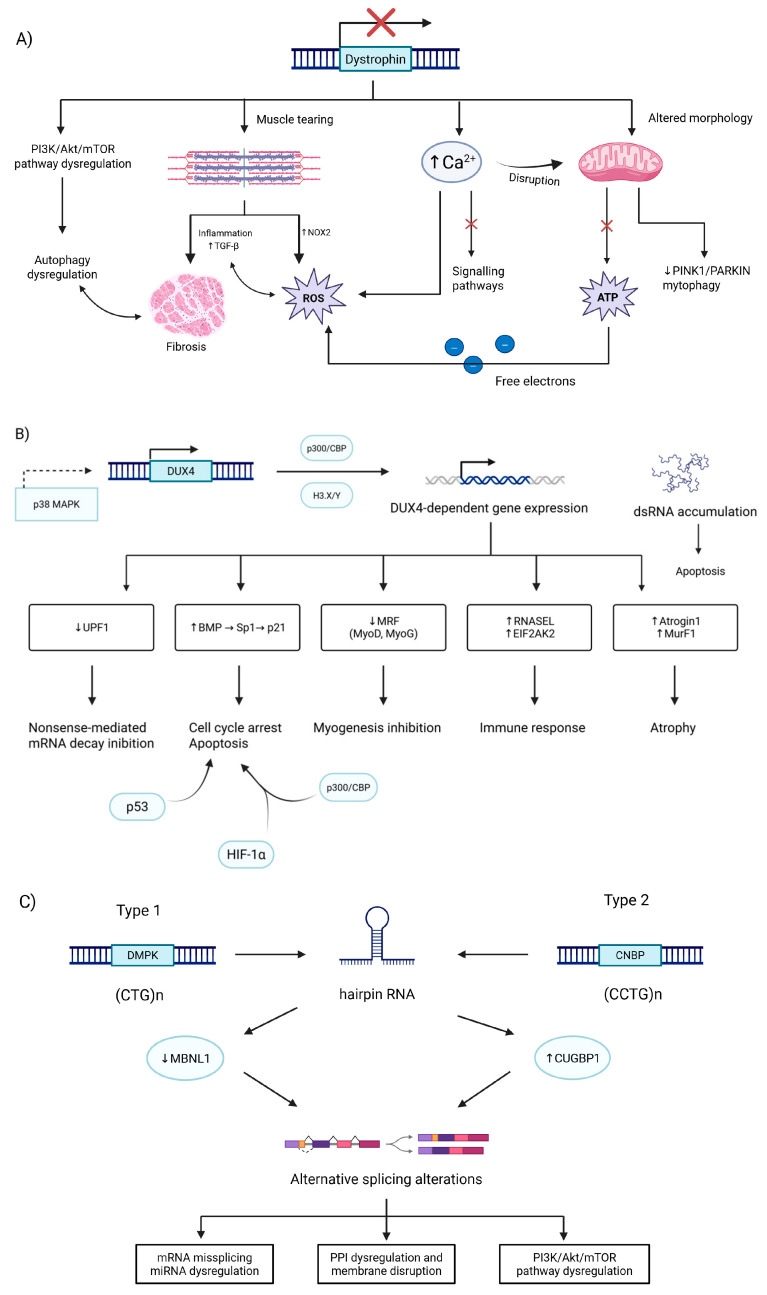
Molecular mechanism of muscular dystrophies. (**A**) Molecular mechanism of Duchenne Muscular Dystrophy. (**B**) Molecular mechanism of Facioscapulohumeral Dystrophy (**C**) Molecular mechanism of Myotonic Dystrophies Type 1 and Type 2. See details in full text. Created in BioRender. Rintz, E. (2025) (**A**) https://BioRender.com/mo27ly5; (**B**) https://BioRender.com/jsm1r9g; (**C**) https://BioRender.com/lmmi6fw.

DMD, as well as BMD, is caused by mutations in the *DMD* gene located on chromosome X, which encodes dystrophin (Figure 6). Occurring mutations, including a variety of frameshifting mutations, prevent the production of the muscle isoform of dystrophin (Dp427m), resulting in a progressive loss of muscle tissue, due to their sensitivity to impairment. As the largest known gene in the human body, DMD has a significant mutation rate that explains a wide spectrum of the disease phenotype [242,243,244,245].

Myotonic dystrophy type 1 results from a trinucleotide CTG repeat expansion in the *DMPK* gene (Figure 6), encoding serine-threonine kinase, while myotonic dystrophy type 2 results from tetranucleotide CCTG repeat expansion in the *CNBP* (formerly *ZNF9*) gene, encoding nucleic acid-binding protein. The amount of expanded repeat accumulating in the muscle nuclei can vary between individuals and also play a role in the toxic effect on a cell [240,246].

As for EDMD, there are two main genes associated with the disease. Approximately 40% of patients with EDMD have mutations in *EMD* or *LMNA* genes located on chromosomes X and 1, respectively. Other linked genes are *SYNE1*, *SYNE2*, *FHL1*, and *TMEM43*. Proteins encoded by these genes are the main parts of a structural framework of the nucleus. The loss of the proteins can affect the nucleus integrity, which can impair muscle density and disturb membrane transport [247]. Recent studies suggest that EDMD pathogenesis may also take part in another nuclear envelope protein, NET39, the loss of which results in stretch-induced DNA damage [248].

The molecular pathology of FSHD is associated with *DUX4* gene and the macrosatellite array of tandemly repeated D4Z4 sequence, which carries the gene. Double homebox 4 protein (DUX4) is a transcription factor involved in embryonic development. It can activate the stem cell and germline programs, presumably distinct from adult skeletal muscle, which may result in later dysfunction. Also, it is assumed that DUX4 can induce apoptosis or inhibit the regeneration of the muscle [249,250].

Autophagy is a catabolic mechanism essential for facilitating muscle regeneration by removing toxic substances, proteins, or damaged organelles. Disruption of balance in autophagy leads to muscle atrophy and impaired stem cell proliferation and differentiation [251,252,253]. Studies revealed that in myotonic dystrophy, muscle activation of pathophysiological pathways, including autophagy, apoptosis, and necrosis, is severely promoted. However, the direct molecular mechanism leading to this imbalance remains unknown [254]. Due to constant membrane tears in sarcolemma occurring in the disease, the level of intracellular calcium is elevated. Constant influx of Ca^2+^ results in mitochondrial swelling, which alters mitochondrial bioenergetics [243,255]. Mitochondrial stress induces mitophagy and autophagy, whereas the impairment of these processes results in muscle atrophy and weakness [255]. Studies suggest that the key component involved in muscle atrophy in dystrophies is the PI3K/Akt/mTOR signaling pathway, which regulates autophagy and myoblast differentiation. Perturbation of this pathway results in excessive autophagosome formation, following reduced autophagic flux that leads to an incomplete autophagy process [251,253,254,256]. Conducted studies also highlight the importance of AMPK pathway and transcriptional downregulation due to suppression of FoxOs and TFEB in the autophagy impairment [251,252,256]. Moreover, studies revealed upregulated expression of p62, a marker of impaired autophagy, which is in control of selective autophagy of ubiquitinated cargos [257,258,259]. Oxidative stress and abnormal activation of Ca2+-dependent calpain protease and phospholipase promotes phosphorylation and activation of Akt by PI3 kinase pathway, which results in autophagy inhibition [243]. Animal studies revealed changes in levels of LC3, BNIP3, HSP70, and SQSTM1, proteins involved in autophagic and mitophagic signaling [253,260,261]. However, there is a debating point, as some research showed elevated levels of LC3 in myofibers from DMD patients, whereas some research showed the opposite. LC3 is a late phase marker of autophagy, and its hyperexpression indicates the activity of this process. This might be explained by the diversity of the studies and differences in the approaches [257,258,259]. In the autophagy mechanism, HSP70 and LC3 proteins may create a complex with BAG3 protein that ensures proper degradation [262]. The connection between BAG3 and muscular dystrophies can be found in their association with DCM etiology. Clinical classification distinguishes syndromic dilated cardiomyopathy, which is DMD-related, and non-syndromic dilated cardiomyopathy. The latter is inherited in an autosomal manner and involves several genes: *TTN*, *BAG3*, and *DES*. Pathogenic DMD variants do not co-occur with no syndromic DCM variants [263,264]. A study conducted by Johnson et al. reported a singular case, where BAG3 variants carriers from one family also carried two DMD VUS variants. It is suggested that this variation may alter biophysical properties of the dystrophin role and affect the phenotype [265]. Together, these data demonstrate an essential role for autophagy during muscle regeneration and its potential in muscle dystrophy treatment.

Patients with various types of muscular dystrophy share a few distinct symptoms: contractures, stiffness, progressive muscle weakness, and atrophy. Severity, manifestation, onset, and muscles affected by dystrophy differ between the types of disorder. At first, patients have difficulty with walking and standing up, and over time, symptoms progress and eventually affect the heart and diaphragm [240,243,247,266,267,268]. Due to muscle degeneration, the level of creatine kinase is highly elevated in these patients, so the diagnosis of muscular dystrophy, before genetic testing, relied on this marker. Nowadays, diagnosis is based on molecular testing, EMG, and MRI, with the assessment of the mentioned enzyme [247,267,269]. There is no evidence supporting physical therapy being beneficial for patients suffering from muscular dystrophy. However, some studies suggest that aerobic and muscular exercises may be advantageous for dystrophy patients, but only to some extent [270,271,272,273].

Medical care in muscular dystrophy patients is multidisciplinary. The golden standard for dystrophy treatment are corticosteroids, such as prednisone, deflazacort, and vamorolone [243,269,274]. However, during this treatment, patients suffer from severe side effects; also these drugs have inconsistent efficiency. As the disorder affects numerous organs, therapy strategies need to include cardiac, pulmonary, and orthopedic care [269,274]. To prevent cardiac complications, many patients beside steroids need to take prescribed β-blockers, ACE inhibitors, or angiotensin receptor blockers [269]. Currently, there is no curative treatment for muscular dystrophy. However, advances in gene therapy have provided many opportunities, which may alter the course of the disease. One of the strategies is based on exon skipping. To restore correct reading frame and expression of the dystrophin gene, antisense oligonucleotide drugs are used. Although this strategy raises some questions about its efficacy, a few drugs have already been approved by the FDA [55,269,274]. Another approach is based on in vivo gene correction or replacement. This strategy involves overcoming a significant obstacle, the size of the dystrophin gene. Numerous studies are conducted to find an ideal vector or to downsize the gene. Current approaches focus on micro-dystrophin, recombinant AAV vector, extracellular vesicles, and lipid nanoparticles [55,274,275]. The latter has been investigated in mice as RNA-delivery vectors. LNP interacts with the endosome membrane receptor, initiates breaking of the endosome membrane, and releases RNA into the cytoplasm. This vector is used for the delivery of Cas9 mRNA and sgRNA in the CRISPR-Cas9 system [45]. The variety of PAM sequences, to which the CRISPR system connects, became a handicap for finding the best possible sgRNA to deliver. To overcome this obstacle, Walton et al. developed PAM-relaxed Cas9 variants, which solved the compatibility limitation issue [276,277,278]. Currently, there are no CRISPR-Cas9-based clinical trials for muscular dystrophy; they are in the planning stages. However, other strategies, including micro-dystrophin and exon skipping are already undergoing phase I–III clinical trials (clinicaltrials.gov: NCT04240314, NCT04626674, NCT05693142). Under consideration is also cell transplantation therapy, which aims to restore dystrophin expression and enable muscle recovery. Various types of cells are investigated as candidates for the transplantation, such as satellite cells, induced pluripotent stem cells, and mesoangioblasts (MABs). Two types of transplantation are examined, autogenic and allogenic cell transplantation, each having its own advantages and drawbacks. As well as in gene therapy, in this strategy, the main obstacle remains the delivery system [242,275,279]. Nevertheless, cell transplantation is undergoing clinical trials for dystrophy treatment (NCT05126758, NCT05338099).

Despite the tremendous development that modern medicine has undergone, there are still numerous genetic diseases that remain incurable. Currently, the only treatment for muscular dystrophies is symptom relief [269,280]. A lot of research is being carried out to permanently correct the genetic variants that determine the disease. Some of these are already in clinical trials [242]. The first drugs to restore the correct reading frame for the dystrophin gene are available in some countries. One therapy is based on mutant exon skipping using antisense oligonucleotides (AONs). AONs bind to the specific sequences in dystrophin pre-mRNA; during mRNA maturation, the mutant exon is being skipped, resulting in restored production of partially functional dystrophin [241]. Another approach is based on blocking a premature stop codon. Binding the restorative chemical compound (as Ataluren) to ribosomes enables the suppression of the premature stop codon reading, resulting in the production of modified dystrophin. However, these drugs are intended for a small group of patients with a specific genetic variant [241,280]. Another obstacle for researchers to overcome is the selection of the most effective delivery system. Due to the size of dystrophin gene, currently available vectors are not efficient, as they exhibit a limited packaging capacity. Moreover, these vehicles display insufficient muscle transduction capability, so their administration needs to be intramuscular [280]. In addition, gene therapy comes at a high cost (up to several million dollars) and is unaffordable for most patients. However, current advances in muscular dystrophy diagnosis may enable the disease prediction in infancy by the detection of pathogenic variants prior to clinical presentation, allowing an early intervention [269]. It is suggested that improvement in muscle delivery system and gene therapy for muscular dystrophy is achievable within a decade. Moreover, to overcome the limitations of available approaches, combining diverse therapies is recommended [241,269,280].

## 3. Discussion and Conclusions

Autophagy is a fundamental cellular process responsible for maintaining cellular homeostasis, including in muscle cells, by degrading and recycling damaged organelles, misfolded proteins, and other cellular debris. It plays a key role in muscle regeneration, metabolic adaptation, and immune regulation. However, when autophagy becomes dysregulated through inactivation, over-activation, or malfunction, it contributes to the onset and progression of various myopathies.

In the article, we showed the molecular background and therapeutic approaches of all the main types of muscular disorders, including the following: Cardiomyopathies, (with an emphasis on dilated cardiomyopathy), Metabolic myopathies, mitochondrial disorders, Myofibrillar myopathies, Congenital myopathies, Idiopathic inflammatory myopathies, and dystrophies, also explaining how impaired autophagy may contribute to the pathogenesis.

**Figure 7:**
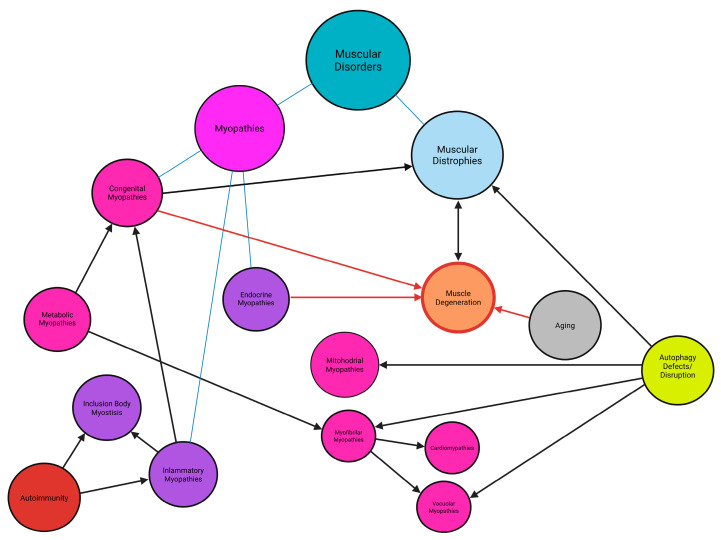
Examples of relationships between main types of myopathies, muscular dystrophies, and autophagy. Arrows—one myopathy or particular process causes another. Created in BioRender. Rintz, E. (2025) https://BioRender.com/80fk48r.

To summarize, highlighting certain relationships between these would be noteworthy (Figure 7). This can be a starting point for discussion about future therapies and possible medical strategies, not only for one particular disease but also for the related ones.

Since autophagy acts as a protective mechanism under physiological conditions, its malfunction can lead to chronic inflammation, mitochondrial dysfunction, impaired lysosomes, and defective muscle regeneration [281]. Intermediate body muscle inflammation causes IBM—interaction between ageing, muscle degeneration, and autoimmunity. Inflammatory myopathy is a chronic condition characterized by progressive muscle weakness, mitochondrial dysfunction, and the accumulation of protein aggregates in muscle fibers. Impaired autophagy plays a dual role in IBM: insufficient autophagic clearance of ubiquitinated proteins and dysfunctional mitochondria accelerates muscle degeneration, while excessive autophagic activity triggers inflammation and activation of the immune system [282]. Recent reports indicate that targeting mitochondrial and lysosomal dysfunction may be crucial to developing a therapeutic strategy for IBM patients [283]. Genetic treatments for IIM aim to promote muscle repair and regeneration, through strategies, such as stem cell or myoblast transplantation, involving genetically corrected cells to restore muscle function. Additionally, stem cell-derived exosomes and growth factors, along with improved viral vector techniques, hold promise for protecting muscles and enhancing tissue repair while minimizing immune responses. On the other hand, hereditary metabolic and endocrine myopathies result from enzyme deficiencies that impair energy production and cellular waste disposal. Dysfunctional autophagy contributes to chronic low-grade inflammation in these conditions, exacerbating muscle damage and fatigue [284,285]. In particular, excessive activation of mTORC1 signaling inhibits autophagy, leading to lipid and glycogen accumulation in metabolic myopathies, such as Pompe disease and McArdle disease. Strategies targeting the regulation of autophagy show the potential to alleviate metabolic stress and increase muscle endurance and in the end, improve the patient’s condition [286]. Defects in autophagy in striated muscle diseases are closely related to vacuolar myopathies, in which autophagic vacuoles accumulate in muscle fibers, disrupting normal muscle function [287]. Furthermore, defective remodeling of lysosomes during autophagy has been shown to lead to skeletal muscle degeneration, highlighting a critical impairment of the endolysosomal pathway [281]. Mitochondrial myopathies that belong to metabolic myopathies, often resulting from mutations affecting oxidative phosphorylation, additionally show impaired autophagy as a key pathogenic factor, with defects in mitophagy leading to persistent oxidative stress and ATP depletion [287,288]. Vitamin and cofactor supplementation, dietary changes, selected aerobic exercises, and arginine can improve mitochondrial function and reduce stroke-like episodes in some of mitochondrial myopathies (especially in MELAS). Myofibrillar myopathies and cardiomyopathies share a common molecular basis due to mutations in key cytoskeleton-building proteins and sarcomeres. These, in turn, are crucial for maintaining the integrity and function of both skeletal and cardiac muscle. Prominent among these are mutations present in the genes encoding desmin and filamin C. These genetic defects contribute specifically to dilated cardiomyopathy and restrictive cardiomyopathy (RCM) by impairing sarcomere stability and mechanical integrity [24]. A hallmark of MFM is the accumulation of abnormally folded proteins that aggregate in muscle cells, including cardiac tissues, exacerbating the deterioration of contraction parameters. These protein aggregates disrupt myofibril organization and impair myocardial contractility, ultimately leading to heart failure in affected individuals [9].

Disease-modifying therapies, such as ACE inhibitors, beta-blockers, MRAs, ivabradine, and cardiac resynchronization, can lead to significant or complete recovery of LV function. Exercise may improve cardiopulmonary health and quality of life, although data specific to DCM is limited. Future treatments should explore targeted approaches addressing the causes of DCM, including autophagy stimulators such as resveratrol, vitamin D3, metformin, caloric restriction, and molecular factors, such as *BEX1*, *RGCC*, *VSIG4*, and HIF-1 signaling. The formation of aggregates is due to the disruption of the autophagy pathway. In addition, activation of matrix metalloproteinases (MMPs), particularly MMP-9, is associated with further muscle degradation, exacerbating damage in both skeletal and cardiac muscle, leading to cardiomyopathies [288]. Myofibrillar myopathies and cardiomyopathies are interconnected: the progressive skeletal muscle damage observed in MFM can also affect the myocardium, also due to the overlapping molecular mechanisms regulating both tissue types (striated tissue, and transverse striated myocardium). As muscle degeneration progresses, cardiomyopathic changes become more prominent, often manifesting as early heart failure or conduction system abnormalities. In addition, oxidative stress also plays a key role in exacerbating disease progression, as overexposure to ROS disrupts normal cellular homeostasis and promotes apoptotic pathways, further impairing both skeletal and cardiac muscle function [24]. MFMs and congenital myopathies share a common genetic landscape, with mutations affecting sarcomeric proteins such as titin, tropomyosin, and desmin. That may also lead to overlapping their phenotypes. These mutations impair myofibril assembly and sarcomeric organization, leading to structural abnormalities, such as Z-line disorganisation, a hallmark of both conditions [85]. In severe cases, mutations in tropomyosin 3 (*TPM3*) and skeletal muscle alpha-actin (*ACTA1*) can result in early forms of MFM that show symptoms similar to those of congenital myopathies [24,289]. The course of MFM in patients in infancy can sometimes be indistinguishable from congenital myopathies, leading to overlapping classifications. Some congenital myopathies, particularly those associated with BAG3 and desmin mutations, show phenotypic similarities to MFM, further complicating the diagnostic process [24]. Furthermore, common mutations in cytoskeletal proteins increase progressive muscle weakness and some congenital myopathies progress to MFM-like disorders later in life [290].

The developmental impact of structural protein mutations also plays a key role in the pathogenesis of congenital myopathies, with some mutations impairing muscle development in utero. While congenital myopathies usually occur after birth, MFMs may appear later in life, exhibiting a spectrum of disease symptoms based on the severity of the underlying genetic mutations [24].

Chronic inflammation causes immune response that can drive to inflammatory myopathies. They can significantly affect muscle repair and regeneration, particularly during fetal development. This progressive immune-mediated muscle damage can also result in the development of congenital myopathies, in which persistent muscle inflammation and oxidative stress impair muscle fiber maturation [291]. This often results in subsequent so-called inflammatory myopathies. In turn, a genetic predisposition to such conditions may increase susceptibility to subsequent inflammation, exacerbating muscle weakness. The molecular and genetic links between myopathies demonstrate the high complexity of muscle disorders. Mutations affecting proteins of the sarcomeres, cytoskeleton, and metabolic pathways result in a continuum of muscle diseases. In these diseases, overlapping phenotypes present a challenge for classification and thus, for the search and implementation of effective therapy. Only by understanding these common mechanisms of the onset and course of these diseases, as well as the positive feedback loops, can we develop targeted therapeutic strategies to mitigate muscle damage and improve patients’ quality of life.

Therefore, there is a rationale for applying shared therapeutic strategies across different myopathies and muscular dystrophies, by adapting elements of treatment for one condition to another [292].

The immune response in inflammatory myopathies can act as a secondary stressor, exacerbating the muscle defects seen in congenital myopathies. Chronic cytokine activation, combined with immune cell infiltration, creates an environment of ongoing muscle damage and impaired regeneration that closely resembles the structural pathology of congenital myopathies [9]. Moreover, congenital myopathies may predispose individuals to inflammatory episodes, as structural muscle defects trigger an immune response, creating a vicious cycle of muscle degradation [293].

Metabolic myopathies and MFM share a common pathogenetic link through defects in energy production that lead to secondary damage to sarcomeres. Mitochondrial dysfunction and glycogen storage abnormalities observed in metabolic myopathies often cause oxidative stress, damaging myofibrils and leading to protein aggregation, a hallmark of MFM [294].

Furthermore, cytoskeletal dysfunction in the MFM may further impair cellular energy pathways, exacerbating mitochondrial stress and metabolic failure [295]. This interaction between energy metabolism and sarcomeric integrity highlights the bidirectional relationship between metabolic myopathies and MFM.

Gene therapy has emerged as a promising and increasingly tailored approach to treating muscular disorders by addressing their underlying genetic causes. These disorders display considerable genetic heterogeneity, necessitating distinct therapeutic strategies (Table 2). In some cases, such as in myofibrillar myopathy type 6 (MFM6), the focus is on silencing the mutant allele to prevent the production of toxic protein variants [149], while in others, such as Pompe disease, therapy aims to restore enzymatic function by delivering a functional gene (e.g., GAA) [296]. In disorders, like Duchenne muscular dystrophy (DMD), the challenge lies in delivering a large, functional protein, such as dystrophin, often requiring truncated or engineered versions suitable for packaging into viral vectors [297]. Across most therapeutic approaches, adeno-associated virus (AAV) vectors have become the platform of choice due to their relative safety, tissue specificity, and ability to mediate long-term gene expression. In cardiomyopathies, such as dilated cardiomyopathy (DCM), various experimental strategies, including RNA interference, antisense oligonucleotides (ASOs), CRISPR, TALENs, and AAV-mediated delivery, are being explored [69,298,299,300], while BAG3-related forms are targeted using allele-specific CRISPR-Cas9 editing in preclinical models [149]. Among metabolic myopathies, AAV-based gene replacement therapies are advancing for Pompe disease [301] with similar AAV-mediated strategies in development for Cori [302] and McArdle diseases [303,304]. Congenital myopathies, such as X-linked myotubular myopathy, are currently being addressed through clinical trials with AAV8-MTM1 (AT132) [NCT03199469], while other forms, like centronuclear myopathy, are targeted via DNM2 silencing approaches and MTM1 gene replacement [175]. In muscular dystrophies, DMD has seen the most clinical progress, with AAV-microdystrophin therapies and exon-skipping agents, like eteplirsen, undergoing clinical use or evaluation [305]. Myotonic dystrophy type 1 (DM1) is being targeted with antisense oligonucleotides, RNA interference, and epigenetic or CRISPR-based strategies, some of which are already in clinical trials (NCT05027269) [306,307]. Facioscapulohumeral dystrophy (FSHD) is being explored using CRISPR interference techniques directed at DUX4 expression, while for Emery–Dreifuss muscular dystrophy, preclinical gene replacement with AAV-Net39 is under study [248]. Inflammatory myopathies, such as inclusion body myositis, are being evaluated in clinical trials using AAV1-Follistatin to enhance muscle mass [308]. Finally, gene therapy research in distal myopathies includes lipoplex-based delivery for GNE myopathy [196] and AAV-based approaches, like SRP-6004, for dysferlinopathies such as Miyoshi myopathy type 2 (NCT05906251). While most of these therapies remain in preclinical or early clinical stages, the growing number of AAV-based strategies tailored to the molecular pathology of each disorder reflects both the complexity and the promise of gene therapy in neuromuscular disease.

Inborn and metabolic myopathies share mutations in mitochondrial and glycogen-related genes, highlighting a common pathogenetic mechanism. Mitochondrial dysfunction during fetal development can impair muscle formation, leading to congenital myopathies after birth [23]. In addition, chronic energy deficiency in utero can disrupt sarcomeric protein assembly, further contributing to congenital muscle weakness [292]. Both metabolic and congenital myopathies also share enzymatic defects, particularly in glycogen storage pathways, which impair cellular energy supply and contribute to muscle fiber dysfunction [239]. This overlap of pathogenetic mechanisms suggests that metabolic dysregulation during early development may contribute to the aetiology of congenital myopathies. Congenital myopathies and muscular dystrophies share genetic mutations in sarcomeric and structural proteins, and some congenital myopathies develop into dystrophic conditions over time. Chronic mechanical stress on muscle fibers in congenital myopathies can trigger degenerative pathways, leading to the dystrophic changes characteristic of progressive muscular dystrophies [309]. In cases, such as myosin-deficient congenital muscular dystrophy, the phenotypic overlap between congenital myopathies and muscular dystrophies become evident as patients show features of both conditions [24]. The presence of progressive structural degeneration in congenital myopathies suggests a continuum of muscle disease, in which early onset conditions may progress to more severe dystrophic phenotypes due to cumulative muscle damage. In other words, the symptoms of congenital myopathies may progress toward muscular dystrophies, which in turn, may predispose to the development of subsequent acquired myopathies [84]. Gene therapy for muscular dystrophy, focusing on micro-dystrophin delivery via AAV vectors or exon skipping with antisense oligonucleotides, is in clinical trials, though challenges remain with gene size and delivery methods, such as liposomes and muscle transduction efficiency. Emerging approaches include CRISPR-Cas9 gene editing, iPSC-derived cells and mesoangioblasts, with both autogenic and allogenic transplantation options being explored. Early diagnosis through genetic testing can enable prediction and intervention before clinical symptoms appear.

The potential of artificial intelligence to revolutionize the diagnosis, monitoring, and treatment of myopathies is increasingly evident, especially through international collaborations and rare disease programs. Notably, the DREAMS consortium, funded by the European Commission with €8 million, aims to develop targeted therapies for five neuromuscular disorders by integrating AI, stem cell research, and pharmacological screening. Complementary initiatives, such as Every Cure, funded by ARPA-H and REPMi4ALL, foster open-source drug repurposing databases and accelerate clinical trials, while platforms, like REPO4EU, facilitate stakeholder collaboration for personalized treatment approaches. AI-driven companies, like HealX and Biovista, leverage machine learning to identify promising existing drugs for rare diseases and neurodegenerative conditions. These collaborative efforts, supported by funding opportunities and innovative pipelines, hold great promise for transforming patient care and overcoming the challenges faced by individuals with myopathies and their families [56].

All of the efforts to improve treatments for rare diseases, including myopathies and dystrophies, require involving a diverse ecosystem of programs, funding sources, and collaborative networks that bridge small foundations with large organizations. These collaborations are crucial because the costs of targeted therapies for a single patient may even exceed USD 2M—if the particular therapy exists. The patients are mostly on their own. However, there are major initiatives, such as the National Organization for Rare Disorders (NORD), the National Institute of Neurological Disorders and Stroke (NINDS), the Wellstone Muscular Dystrophy Cooperative Research Centers, Horizon Europe, European Reference Networks (ERN), and the Rare Diseases Clinical Research Network (RDCRN), that exemplify how coordinated efforts can accelerate research and therapy development. Non-governmental organizations, like the Muscular Dystrophy Association (MDA), Cure Rare Disease, and Parent Project Muscular Dystrophy (PPMD), along with biotech and pharmaceutical companies, like Sarepta Therapeutics, Pfizer, and Roche, sometimes may collaborate with smaller foundations, such as AFM-Téléthon (France), Cure Duchenne (UK), Huntington’s Disease Society of America (HDSA), Charcot-Marie-Tooth (CMT) Research Foundation, Hereditary Spastic Paraplegia (HSP) Foundation (Canada/US), Spinal Muscular Atrophy (SMA) Foundation, TREAT-NMD (European), Global Genes (USA), The Michael J. Fox Foundation (USA), The Batten Disease Support & Research Association (BDSRA), BAG3 Research Foundation (Pl), Cure HSPB8 (Global), the HSP Research Foundation, and the universities. All these foundations, research facilities and other mentioned before institutions, focus on the very same goal, which makes therapies as accessible as possible.

These collaborations can facilitate shared expertise, funding, and resources to design and optimize targeted therapies—particularly gene therapies and AAV technologies—while also addressing critical issues, such as lowering costs and shortening development timelines. Since rare diseases have limited commercial potential, bypassing lengthy patenting procedures and licensing barriers can expedite treatment availability, especially through non-profit models. Larger industry partners benefit by expanding their technological portfolio, enhancing the visibility of their projects, and refining their existing gene editing and viral vector platforms with less emphasis on immediate profits but broader therapeutic impact. This synergy between big and small players is vital to delivering faster, more accessible, and cost-effective treatments to patients worldwide. Small foundations and patient groups often collaborate with academia and larger programs by sharing data, participating in laboratory research, and clinical trial design, as well as advocating for regulatory support. They can expedite treatment development by focusing on niche research areas, fostering innovative therapies, and supporting early-stage research. Larger organizations can help bypass costly patent procedures and licensing barriers by endorsing open-access models or non-profit-based drug development, especially when commercial interests are limited due to the small number of patients. Additionally, collaboration can enhance resource sharing, speed up regulatory approval, and lower costs, ultimately creating a more efficient pipeline from discovery to patient access.

**Table 2:** Representative subtypes of each discussed muscular. Cardiomyopathy: DCM; Metabolic myopathies: GSD II, GSD III, GSD V, CPT II deficiency, MELAS; Congenital Myopathy: MTM1-associated myopathy, CCD, MmD, Nemaline myopathy, CNM; Idiopathic inflammatory myopathies: DM, PM, IBM, IMNM; Myofibrillar myopathies: MFM1–MFM6; Distal Myopathy: Welander distal myopathy, Tibial (Udd) myopathy, GNE, MMD2; Muscular Dystrophy: DMD, DM1, FSHD, EDMD.

Subtype	Gene(s) Involved	Pathways	Clinical Phenotype	Current Therapies	Experimental Gene Therapy Approaches	Ref.
Dilated (DCM)	*TTN*, *PLN*, *MYH7*, *BAG3*, (frequent variants shown)	PI3K/Akt, NADPH/ROS, MAPK, PLC/IP3/Ca/NFATFAS-dependent apoptosis pathway	Arrhythmias, dyspnea, edema, thromboembolis, cardiac dilation, fibrosis, muscle wasting	ACE inhibitors, ARBs, β-blockers, MRAs, ivabradine; CRT; aerobic and resistance training; cardiac rehab	RNAi, ASO, CRISPR, TALEN, AAV vectors	[8,62,69,84,298,299,300]
Pompe disease (GSD II)	GAA	Lysosomal glycogen degradation; autophagy (mTORC1 regulation)	Infantile cardiomyopathy, hypotonia, respiratory failure; late-onset: proximal muscle weakness, fatigue; elevated CK	Enzyme replacement therapy (alglucosidase alfa); exercise restriction; nutritional support; emerging small-molecule chaperones (e.g., miglustat)	AAV-mediated GAA gene therapy in development	[87,99,101,104,105,310]
Cori disease (GSD III)	AGL	Glycogen debranching enzyme activity	Hepatomegaly, hypoglycemia (juvenile forms), myopathy; muscle weakness and fatigue; hepatic cirrhosis in severe cases	High-protein, frequent meals (uncooked cornstarch); monitoring of liver function; experimental liver-targeted gene therapy not yet available (no ERT)	AAV Vector Encoding a Bacterial Glycogen Debranching Enzyme	[95,97,106,302,310]
McArdle disease (GSD V)	PYGM	Muscle glycogen phosphorylase (glycogenolysis)	Exercise-induced cramps, myalgia, fatigue; “second wind” phenomenon (improved tolerance after brief rest); normal life expectancy with management	Pre-exercise simple sugars (glucose) or sucrose; regular moderate exercise; pain management; creatine supplementation (investigational)	rAAV8-Pygm improving glycogenolysis and muscle performance; AAV delivery of PYGM cDNA to ovine muscles; systemic rAAV8-Pygm delivery in murine model	[107,108,110,111,303,304]
CPT II deficiency	CPT2	Mitochondrial fatty acid β-oxidation (entry into mitochondria)	Episodic myalgia and rhabdomyolysis triggered by prolonged exercise/fasting; mild fixed myopathy (adult form); episodes of myoglobinuria; normal CK between attacks	Prevent fasting/excess exercise; high-carbohydrate diet, medium-chain triglycerides; L-carnitine supplement; riboflavin in some cases	–	[31,112,113,115,116]
MELAS (m.3243A>G)	MT-TL1 (mitochondrial tRNA Leu(UUR))	Oxidative phosphorylation (respiratory chain complexes); mitochondrial energy metabolism	Stroke-like episodes (neurologic deficits), seizures, migraines; lactic acidosis; progressive myopathy and exercise intolerance; cardiomyopathy; short survival (median ~17 yr post-onset)	Symptomatic: arginine for stroke-like episodes; coenzyme Q10, L-carnitine, vitamins (B complexes); exercise training; diet modifications	mitoARCUS nuclease for elimination of MELAS-associated m.3243G mtDNA	[86,120,121,122,123,311]
MTM1-associated myopathy	*MTM1*	PI3P metabolism, Dysregulated autophagy	Severe muscle weakness, atrophy of fibers, disrupted membrane trafficking, accumulation of cellular debris, reduced lifespan	AT132 (AAV-MTM1 gene therapy), Non-invasive ventilation, tracheostomy/mechanical ventilation (if needed), physical therapy, nutritional support, multidisciplinary care	AT132 (AAV8-MTM1)	[169,170,171,172]
Central core disease (CCD)	*RYR1*	Calcium dysregulation (SR Ca^2+^ release)	Single central core lesions, hypotonia, muscle weakness, increased risk of malignant hyperthermia	Avoidance of malignant hyperthermia-triggering agents (e.g., volatile anesthetics, succinylcholine), physical therapy, monitoring for cardiac or orthopedic issues	–	[158,159,167,168]
Multiminicore disease (MmD)	*SEPN1*	Oxidative stress, Calcium homeostasis	Multiple small cores in fibers, progressive muscle weakness, respiratory involvement	Early and often intensive respiratory support, physical therapy, management of scoliosis and thoracic deformities, pulmonary care	–	[89,159,160,161]
Nemaline myopathy	*NEB*, *TPM3*, *ACTA1*, *KLHL40*, *KLHL41*, *KBTBD13*	thin filament assembly), Ubiquitin–proteasome degradation	Rod-like (nemaline) inclusions in muscle, muscle weakness, dysmorphic facial features	Physical therapy, respiratory support, nutritional support	–	[157,160,162,163]
Centronuclear myopathy (CNM)	Autosomal: *DNM2,* *BIN1, RYR1,* X-linked: *MTM1*	Membrane trafficking, PI3P metabolism, T-tubule defects	Centralized/internalized nuclei, muscle weakness, t-tubule disorganization	Physical therapy, respiratory support, nutritional support, monitoring for orthopedic and pulmonary complications	rAAV-shDnm2 (preclinical), AAV8-MTM1 for X-linked MTM	[158,159,170,175]
Dermatomyositis (DM)	– And autoantigens: Mi-2, NXP-2, TIF1γ	Complement-mediated microangiopathy, Type I IFN signaling	Proximal symmetrical weakness, skin rash (Gottron’s papules, heliotrope rash), dysphagia, ILD, elevated CK	Immunosuppression (steroids, methotrexate, azathioprine), IVIG (especially for refractory cases), antimalarials for skin rash, exercise therapy (aerobic, resistance); rituximab (RTX) for refractory cases	–	[203,204,205,213,214]
Polymyositis (PM)	–	CD8^+^ T-cell–mediated muscle fiber damage, MHC class I upregulation	Symmetrical proximal muscle weakness, no rash, dysphagia, ILD, elevated CK	Corticosteroids, steroid-sparing immunosuppressants (azathioprine, mycophenolate, methotrexate), exercise therapy (aerobic and resistance), rituximab for refractory cases	–	[204,205,218,219]
Inclusion-body myositis (IBM)	*LDB3/Z* And autoantigens ASP,	Mixed inflammatory (CD8^+^ T-cell–mediated) and degenerative processes involving β-amyloid and tau accumulation, MHC class I upregulation, granzyme B cleaves FHL1, and resulting fragments become autoantigens	Insidious onset quadriceps and finger flexor weakness, muscle biopsy shows rimmed vacuoles, β-amyloid inclusions, poor response to immunotherapy	No proven therapy, poor response to immunosuppressants, IVIG may help some, exercise improves function	AAV1-Follistatin (clinical trial)	[136,147,205,216,226]
Immune-mediated necrotizing myopathy (IMNM)	*HMGC, SRP*	Necrotizing (myocytolytic) muscle pathology; complement activation; anti-HMGCR or anti-SRP autoantibody–mediated injury	Rapidly progressive severe weakness, very high CK, necrosis on biopsy, can be statin-associated (HMGCR)	Corticosteroids; steroid-sparing agents (e.g., azathioprine), IVIG (effective in anti-HMGCR cases), rituximab (RTX) for refractory cases	–	[204,205,220]
MFM1 (Desmin myopathy)	DES	Intermediate filament network (Z-disk scaffold); protein quality control	Progressive distal and proximal weakness; cardiomyopathy; cores and inclusions on biopsy	Supportive (physical therapy, cardiac care)	Single high-dose AAV9 carrying human DES under a cardiac promoter	[129,130,131,132,144,312]
MFM2 (αB-crystallin myopathy)	CRYAB (HSPB5)	Small heat-shock protein (chaperone); aggregates clearance	Progressive myopathy with early cataracts; weakness often starts distal; cardiac involvement possible	Supportive; cataract management	–	[133,313]
MFM3 (Myotilin myopathy)	MYOT	Z-disk assembly (myotilin-actinin-filamin C complex)	Late-onset proximal and distal weakness; myalgia; cardiomyopathy in some; protein aggregates on biopsy	Supportive; manage cardiomyopathy	AAV6 vectors expressing microRNAs targeting the mutant MYOT transcript	[135,314]
MFM4 (LDB3/ZASP myopathy)	LDB3 (ZASP)	Z-disk integrity (actin-binding); autophagy (CASA complex)	Distal and proximal muscle weakness; cardiomyopathy; protein aggregates (ZASP, filamin, Hsp70)	Supportive; exercise, cardiac care	AAV vectors carrying shRNA/miRNA against mutant LDB3	[136,315]
MFM5 (Filamin C myopathy)	FLNC	Filamin C cross-linking (actin-binding); autophagy (CASA)	Adult-onset distal/proximal weakness; cardiomyopathy common; protein aggregates (FLNC, CASA proteins)	Supportive; monitor heart; no cure yet (future: modulate autophagy)	CRISPR/Cas9 strategy for the generation of a FLNC knockout hESC line	[316,317]
MFM6 (BAG3 myopathy)	BAG3	Chaperone-assisted selective autophagy (CASA complex)	Childhood-onset distal/proximal weakness, rigid spine, scoliosis; early cardiomyopathy; elevated CK	Supportive (ventilation, cardiac care)	Personalized allele-specific CRISPR-Cas9 (preclinical)	[11,138,139,140,142,149]
Welander distal myopathy	*TIA1*	Impaired stress granule assembly (TIA1), disrupted RNA metabolism, mRNP accumulation and translation inhibition	Early weakness of thumb/index finger, difficulty extending fingers; later, tibialis anterior weakness, foot drop, steppage gait	Supportive (orthoses for hand/foot drop)	–	[178]
Tibial (Udd) myopathy	*TTN*	Disrupted sarcomeric structure, impaired muscle elasticity, altered protein-protein interactions (e.g., calpain-3, α-actinin, myosin), Progressive muscle fiber degeneration	Adult-onset foot dorsiflexor weakness, slow progression, often spares upper limbs	Supportive (ankle-foot orthoses); physical therapy, cardiac surveillance (titin variants)	–	[179,180]
GNE myopathy (Nonaka)	*GNE*	Impaired membrane repair (dysferlin deficiency), dysregulated Ca^2+^ signaling, enhanced phagocytosis in monocytes, accumulation of necrotic/regenerating fibers and fibrotic tissue	foot drop, sparing of quadriceps, hand/finger flexor weakness, respiratory function preserved	Exercise, L-carnitine, sialic acid precursor supplements (Ace-ER—mixed results)	GNE lipoplex (preclinical)	[180,181,183,184,185,194,196]
Miyoshi Myopathy Type 2 (MMD2)	*DYSF*	Membrane repair dysfunction, Vesicle fusion defects, T-tubule development and Ca^2+^ signaling disruption	Calf/thigh weakness and atrophy, progression to upper limbs, very high CK levels, necrosis, regeneration, fat/connective tissue replacement	Supportive care (physical therapy, orthoses)	SRP-6004 (clinical trial) NCT05906251	[181,183,196,197,198]
Duchenne muscular dystrophy (DMD)	DMD	Sarcolemmal stability (dystrophin-glycoprotein complex)	Childhood-onset progressive proximal weakness, loss of ambulation by teens; dilated cardiomyopathy; scoliosis; elevated CK (~10–100× normal)	Corticosteroids; cardiac care (ACEI/β-blockers);	AAV-microdystrophin and exon-skipping (eteplirsen, golodirsen) in clinical use/trials	[243,244,245,318]
Myotonic dystrophy type 1 (DM1)	DMPK	RNA toxicity (toxic CUG-expanded RNA sequesters splicing factors)	Adult-onset distal and facial weakness, myotonia, cataracts, cardiac conduction defects; multisystem (insulin resistance); progressive	Symptomatic: mexiletine for myotonia; pacemakers for heart block; modafinil for fatigue; cognitive therapies; experimental antisense oligonucleotides (ATX-01)	siRNA (siRNA-CAG)—mutant DMPK mRNA degradation (preclinical); AOC-1001 (antibody-oligo conjugate)—DMPK mRNA knockdown (Phase I/II recruiting) (NCT05027269); SaCas9- or eSpCas9-paired sgRNAs (rAAV9), dSaCas9-KRAB (rAAV6/9) for CTG repeat excision or transcriptional repression (preclinical)	[246,269,306,307,319,320,321]
Facioscapulohumeral dystrophy (FSHD)	DUX4 (permissive 4q allele with D4Z4 contraction)	Misexpression of embryonic DUX4 transcription factor; epigenetic de-repression	Variable adult-onset weakness (facial, scapular, humeral muscles); scapular winging; foot drop; CK normal; slow progression	Supportive: physical therapy, scapular fixation surgery	Epigenetic CRISPRi (dSaCas9-KRAB) targeting DUX4 promoter	[249,250,269,322]
Emery–Dreifuss muscular dystrophy (EDMD)	EMD or LMNA (X-linked or AD); others (SYNE1/2, FHL1)	Nuclear envelope integrity; mechanical stability of nucleus	Early contractures (elbows/Achilles); humeroperoneal muscle weakness; cardiomyopathy with conduction defects; long QT	Pacemaker/ICD for conduction/cardiomyopath; ACE inhibitors; physical therapy for contractures	AAV-Net39 gene replacement (preclinical)	[248,269,274]

## Figures and Tables

**Table 1:** Division of IIMs into types and respective subtypes with general symptoms. Acronyms: PM—polymyositis, DM—dermatomyositis, IMNM—immune-mediated necrotizing myopathy, NAM—necrotizing autoimmune myopathy, HMGCR—3-hydroxy-3-methylglutaryl-CoA reductase, SRP—signal recognition particle, OM—overlap myositis, ASS—anti-synthetase syndrome, IBM—inclusion body myositis, CTD—connective tissue disorder.

Type	PM	DM	IMNM/NAM	OM	IBM
Subtypes	unknown	juvenile DM	statin-associated HMGCR antibody NAM	ASS	unknown
		myopathic DM	statin-naïve HMGCR antibody NAM		
		DM sine dermatitis	SRP antibody NAM		
Symptoms		- pathognomonic skin changes of Gottron’s papules - heliotrope rash - cutaneous changes	- atrophy in affected muscles - weight loss - necrosis on biopsy - cardiorespiratory complications	- systemic lupus erythematosus - rheumatoid arthritis - scleroderma - associated with underlying CTD	- loss of finger dexterity - forearm medial compartment flexors and quadriceps atrophy - muscle involvement more asymmetric than in other IIMs (usually the non-dominant arm more affected)
Overall comparison		- proximal weakness - higher CK			- long finger flexors and quadriceps - normal/moderately higher CK

## Data Availability

Not applicable.

## Abbreviations

The following abbreviations are used in this manuscript:

DCM	dilated cardiomyopathy
HF	heart failure
ACEI	angiotensin-converting enzyme inhibitor
ARB	angiotensin II receptor blocker
MRA	mineralocorticoid receptor antagonist
GSD	glycogen storage diseases
FAO	fatty acid oxidation
ERT	enzyme replacement therapy
GSDs	glycogen storage diseases
GAA	acid alpha-glucosidase
GSD II	Pompe disease
GSD III	Cori disease
GSD IV	Andersen disease
GSD V	McArdle disease
rhGAA	recombinant human GAA
M6P	mannose-6-phosphate
AGL	glycogen debranching enzyme
G6P	glucose-6-phosphate
CK	creatine kinase
MADD	multiple acyl-CoA dehydrogenase deficiency
LCFA	long-chain fatty acids
VLCFA	very long-chain fatty acids
NGS	next-generation sequencing
mtDNA	mitochondrial DNA
PCD	primary carnitine deficiency
CPT I	carnitine palmitoyltransferase I
CPT II	carnitine palmitoyltransferase II
RRFs	ragged red fibers
MELAS	mitochondrial encephalopathy with lactic acidosis and stroke-like episodes
MERRF	myoclonic epilepsy with ragged red fibers
ROS	reactive oxygen species
glucose 1-P	glucose-1-phosphate
MFMs	myofibrillar myopathies
HspB5	alpha-crystalin B chain
CASA	chaperone-assisted selective autophagy
CMs	congenital myopathies
CCD	central core disease
MmD	minicore disease
CFTD	congenital fiber type disproportion
ActRIIB	activin receptor type IIB
MMD2	Miyoshi myopathy type 2
MMD1	Miyoshi myopathy type 1
EMG	electromyography
IIMs	idiopathic inflammatory myopathies
LMOD3	leimodin-3
NRAP	nebulin-related anchoring protein
CUL3	Cullin-3
PI3P	phosphatidylinositol 3-phosphate
mRNPs	messenger ribonucleoprotein complexes
mTORC1	mammalian target of rapamycin complex 1
AAV	adeno-associated virus
TLRs	toll-like receptors
DAMPs	damage associated with molecular patterns
MFM4	Markesbery-Griggs distal myopathy
CAGR	compound annual growth rate
NF-κB	nuclear factor kappa B
DMRVs	distal myopathy with rimmed vacuoles
GNE myopathy	Nonaka myopathy
MRI	magnetic resonance imaging
CT	computed tomography
AI	artificial intelligence
LVEF	left ventricular ejection fraction
GPCR	G-protein-coupled receptor
rAAAV8	recombinant AAV serotype 8
LAMP1	lysosome membrane-associated protein 1
LAMP2	lysosome membrane-associated protein 2
shRNA	small hairpin RNA
PM	polymyositis
IMNM	immune-mediated necrotizing myopathy
NAM	necrotizing autoimmune myopathy
HMGCR	3-hydroxy-3-methylglutaryl-CoA reductase
SRP	signal recognition particle
OM	overlap myositis
ASS	anti-synthetase syndrome
IBM	inclusion body myositis
CTD	connective tissue disorder
MSAs	myositis-specific antibodies
MHC-1	Major Histocompatibility Complex 1
DM	dermatomyositis
IVIG	intravenous immunoglobulin
ADL	activities of daily living
ZASP	Z-band alternatively spliced PDZ-motif protein
FHL1	four-and-a-half LIM domain 1
ILD	interstitial lung disease
RTX	rituximab
rAAV1	recombinant AAV serotype 1
TEEs	thromboembolic events
MSCs	mesenchymal stem cells
iPSCs	induced pluripotent stem cells
DMD	Duchenne muscular dystrophy
BMD	Becker muscular dystrophy
FSHD	facioscapulohumeral dystrophy
EDMD	Emery-Dreifuss muscular dystrophy
AONs	antisense oligonucleotides
MABs	mesoangioblasts
DUX4	double homebox 4 protein
NORD	National Organization for Rare Disorders
MMPs	matrix metalloproteinases
RCM	restrictive cardiomyopathy
NINDS	National Institute of Neurological Disorders and Stroke
RDCRN	Rare Diseases Clinical Research Network
ERN	European Reference Networks
PPMD	Parent Project Muscular Dystrophy
MDA	Muscular Dystrophy Association
Ace-ER	acenouramic acid
HSP	hereditary spastic paraplegia
BDSRA	The Batten Disease Support & Research Association
CMT	Charcot-Marie-Tooth
HDSA	Huntington’s Disease Society of America
SMA	spinal muscular atrophy
LDB3	LIM domain binding 3
